# Supplementary material for: Factors associated with psychological distress during the coronavirus disease 2019 (COVID-19) pandemic on the predominantly general population: A systematic review and meta-analysis
Source: PLoS One. 2020 Dec 28;15(12):e0244630. doi: 10.1371/journal.pone.0244630 (PMC7769562; doi:10.1371/journal.pone.0244630)
Supplement: S1 File — (DOCX) [file pone.0244630.s002.docx]

**Factors associated with psychological distress during the coronavirus disease 2019 (COVID-19) pandemic on the general population: a systematic review and meta-analysis**

Yeli Wang, Monica Palanichamy Kala, Tazeen Hasan Jafar

**The following materials are included in the Online Supplemental Material:**

**Search strategies.**

**S1 Fig. Forest plot of the association between gender (women vs. men) and secondary outcomes of psychological distress.**

**S2 Fig. Forest plot of the association between age (younger vs. older) and secondary outcomes of psychological distress.**

**S3 Fig. Forest plot of associations between education (lower vs. higher), income (lower vs. higher), current employment (yes vs. no) and secondary outcomes of psychological distress.**

**S4 Fig. Forest plot of the association between having higher risk of COVID-19 infection and anxiety and depression.**

**S5 Fig. Forest plot of the association between having higher risk of COVID-19 infection and secondary outcomes of psychological distress.**

**S6 Fig. Forest plot of media exposure (longer vs. shorter), social support (yes vs. no), physical activity (longer vs. shorter) and anxiety and depression.**

**S7 Fig. Forest plot of the association between media exposure (longer vs. shorter) and secondary outcomes of psychological distress.**

**S8 Fig. Potential publication bias tested by funnel plots on gender, age, and socioeconomic status (education, income) with anxiety and depression.**

**S1 Table. Assessment of study quality using Joanna Briggs Institute scores for studies included in the meta-analysis.**

**S2 Table. Stratified analyses of factors and risks of anxiety and depression by study locations.**

**S3 Table. Sensitivity analyses of factors and risks of anxiety and depression among studies using the same instrument and cut-off values.**

**S4 Table. Sensitivity analyses of factors and risks of anxiety and depression among studies excluding patients and healthcare professionals.**

**S5 Table. The attributable risk of depression due to the COVID-19 pandemic.**

**Search strategies**

(Dec 2019 to 15 July 2020)

**PubMed:**

- (psycholog* OR mental OR anxiety OR depression OR fear OR stress OR distress OR post-traumatic OR insomnia OR somatisation OR somatization OR "Stress, Psychological"[Mesh] OR "Psychological Distress"[Mesh] OR "Mental Disorders"[Mesh] OR "Depression"[Mesh] OR "Anxiety"[Mesh] OR "Stress Disorders, Post-Traumatic"[Mesh] OR "Fear"[Mesh] OR "Sleep Initiation and Maintenance Disorders"[Mesh] OR "Somatoform Disorders"[Mesh]) AND (covid-19 OR covid OR 2019-ncov OR sars-cov-2 OR novel coronavirus OR "severe acute respiratory syndrome coronavirus 2" [Supplementary Concept] or "COVID-19" [Supplementary Concept])

**EMBASE:**

- ('psycholog*' OR 'mental disease'/exp OR 'mental disease' OR 'mental health'/exp OR 'mental health' OR 'behavior'/exp OR 'behavior' OR 'mental' OR 'anxiety' OR 'anxiety'/exp OR 'depression' OR 'depression'/exp OR 'fear' OR 'fear'/exp OR 'stress' OR 'stress'/exp OR 'distress'/exp OR 'distress' OR 'post-traumatic' OR 'post-traumatic'/exp OR 'insomnia' OR 'insomnia'/exp OR 'somatisation' OR 'somatisation'/exp OR 'somatization' OR 'somatization'/exp) AND ('covid 19'/exp OR 'covid 19' OR 'covid-19' OR covid OR '2019 ncov' OR 'sars cov 2' OR 'novel coronavirus' OR 'severe acute respiratory syndrome coronavirus 2'/exp OR 'severe acute respiratory syndrome coronavirus 2') AND [embase]/lim

**SCOPUS**

- TITLE-ABS-KEY ((psycholog* OR mental OR anxiety OR depression OR fear OR stress OR distress OR post-traumatic OR insomnia OR somatisation OR somatization ) AND ( covid-19 OR "novel coronavirus" OR covid OR "2019 nCoV" OR "sars cov 2" OR "'severe acute respiratory syndrome coronavirus 2"))

**WHO COVID-19 Database**

- (tw:(psycholog*)) OR (tw:(mental)) OR (tw:(anxiety)) OR (tw:(depression)) OR (tw:(fear)) OR (tw:(stress)) OR (tw:(distress)) OR (tw:("post-traumatic")) OR (tw:(insomnia)) OR (tw:("somatisation")) OR (tw:("somatization"))

**Cochrane Library**

- - 1. #1 ("psycholog*"):ti,ab,kw
    2. #2 ("mental"):ti,ab,kw
    3. #3 ("anxiety"):ti,ab,kw
    4. #4 ("depression"):ti,ab,kw
    5. #5 ("fear"):ti,ab,kw
    6. #6 ("stress"):ti,ab,kw
    7. #7 ("distress"):ti,ab,kw
    8. #8 ("post traumatic"):ti,ab,kw
    9. #9 ("insomnia"):ti,ab,kw
    10. #10 ("somatisation disorder"):ti,ab,kw
    11. #11 ("somatization disorder"):ti,ab,kw
    12. #12 MeSH descriptor: [Mental Disorders] explode all trees
    13. #13 ((COVID-19 OR COVID OR nCoV-2019 OR "novel coronavirus" OR "severe acute respiratory syndrome 2" OR sars-cov-2)):ti,ab,kw (Word variations have been searched)
    14. #14 (#1 OR #2 OR #3 OR #4 OR #5 OR #6 OR #7 OR #8 OR #9 OR #10 OR #11 OR #12) AND #13

**PsycINFO**

- (COVID-19 or COVID or nCoV-2019 or "2019 nCoV" or "novel coronavirus" or sars-cov-2 or "severe acute respiratory syndrome 2").mp.

**S1 Fig.** **Forest plot of the association between gender (women vs. men) and secondary outcomes of psychological distress.**


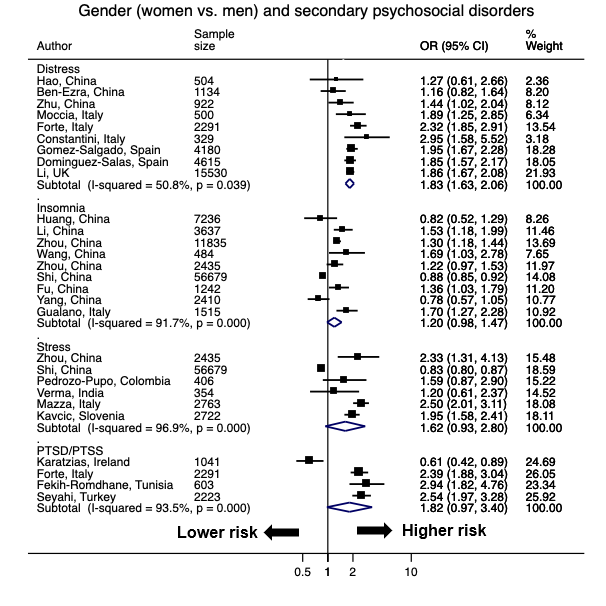


**Legend:** The size of the data markers indicates the weight of the study, which is the inverse variance of the effect estimate. The diamond data markers indicate the pooled ORs.

**Abbreviations:** PTSD/PTSS, post-traumatic stress disorder/symptoms.

**
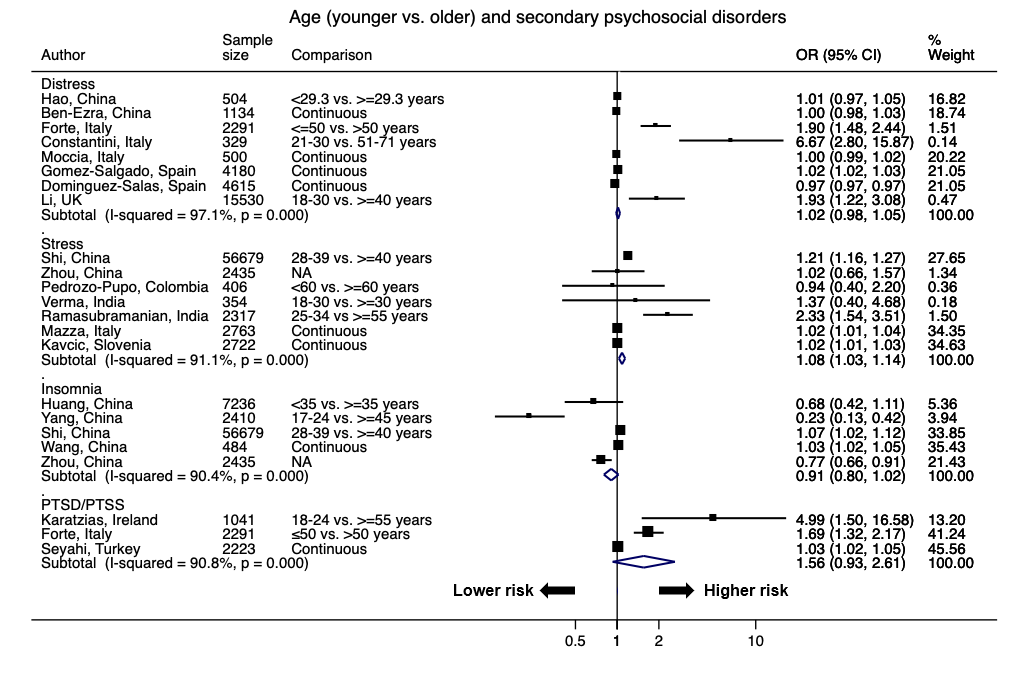
****S2 Fig. Forest plot of the association between age (younger vs. older) and secondary outcomes of psychological distress.**

**Legend:** The size of the data markers indicates the weight of the study, which is the inverse variance of the effect estimate. The diamond data markers indicate the pooled ORs.

**Abbreviations:** PTSD/PTSS, post-traumatic stress disorder/symptoms.

**S3 Fig. Forest plot of associations between education (lower vs. higher), income (lower vs. higher), current employment (yes vs. no) and secondary outcomes of psychological distress.**


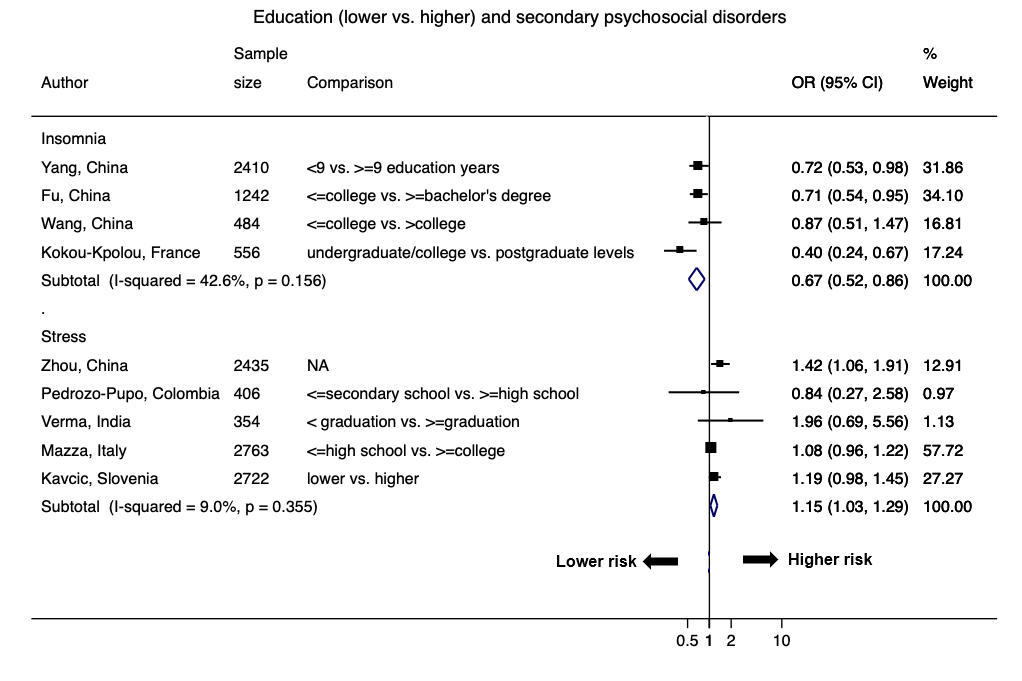


**A)**

**
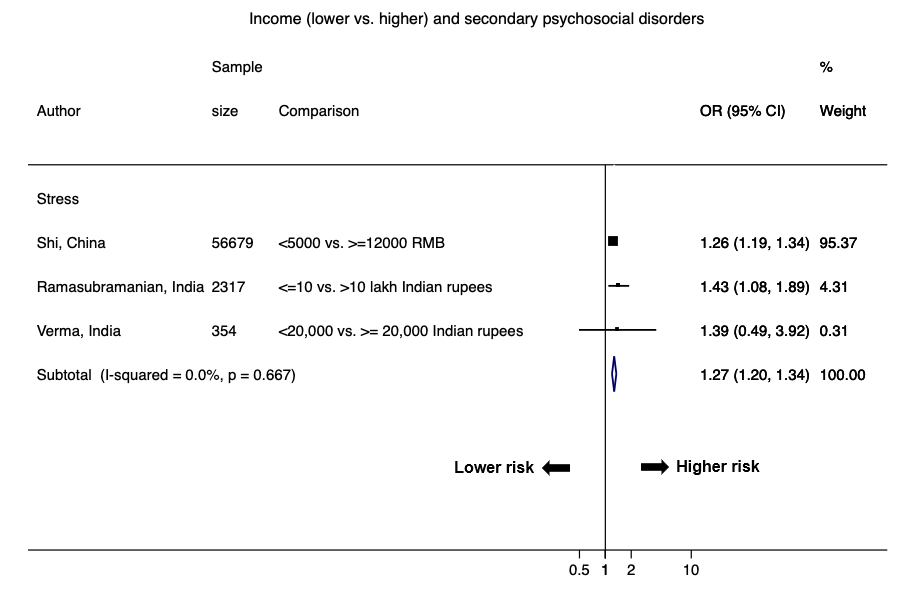
**

**B)**

**
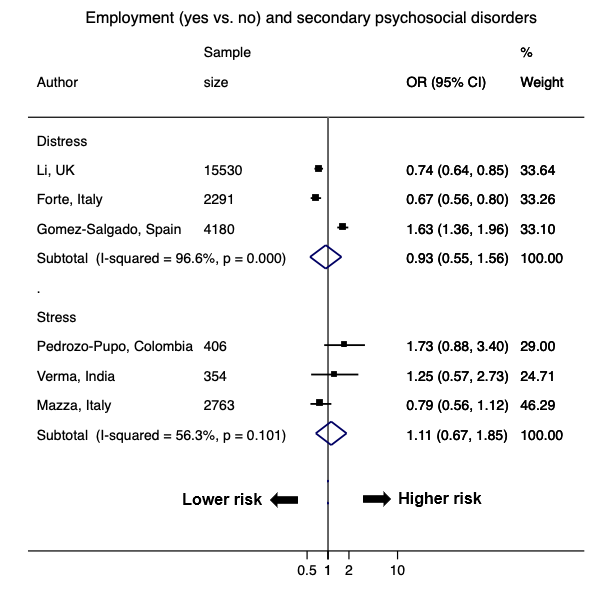
**

**C)**

**Legend:** The size of the data markers indicates the weight of the study, which is the inverse variance of the effect estimate. The diamond data markers indicate the pooled ORs.

**
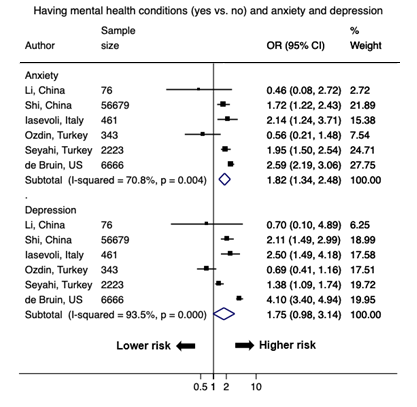

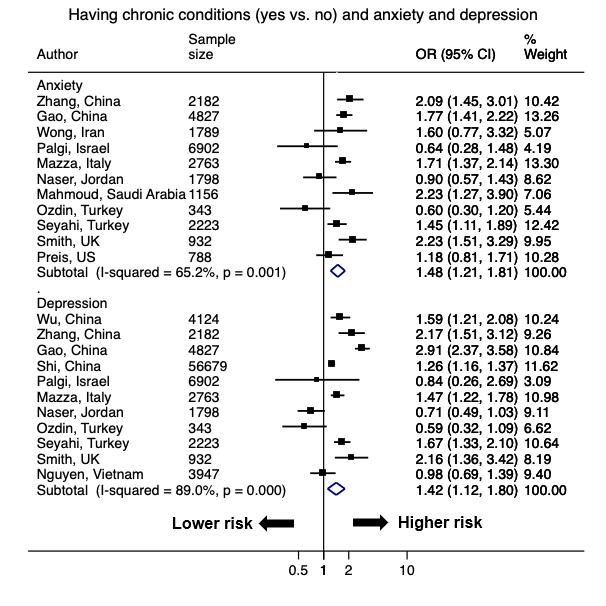

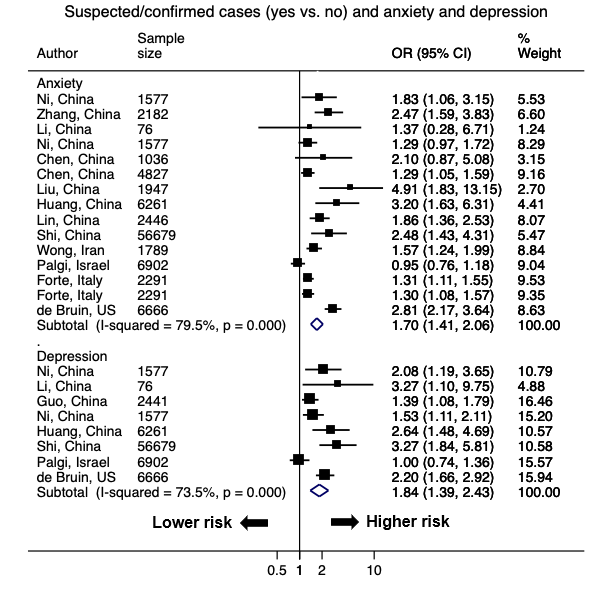

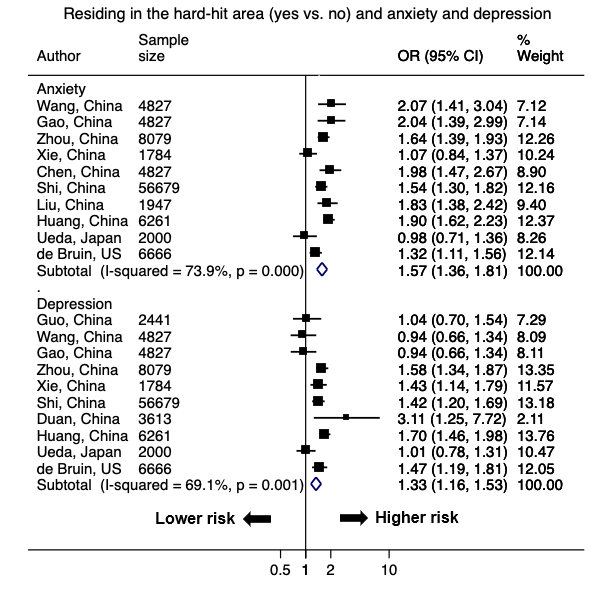
S4 Fig.** **Forest plot of the association between having higher risk of COVID-19 infection and anxiety and depression.**

**D)**

**C)**

**B)**

**A)**

**Legend:** The size of the data markers indicates the weight of the study, which is the inverse variance of the effect estimate. The diamond data markers indicate the pooled ORs.

**S5 Fig. Forest plot of the association between having higher risk of COVID-19 infection and secondary outcomes of psychological distress.**

**A)**

**
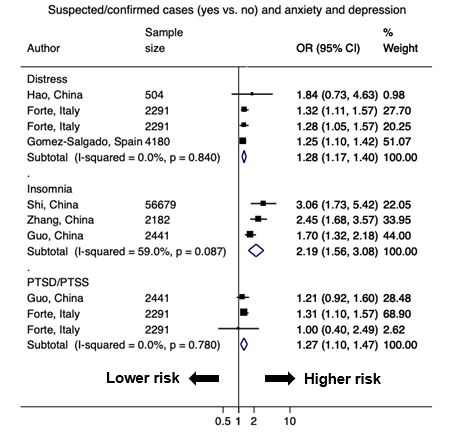
**

**B)**


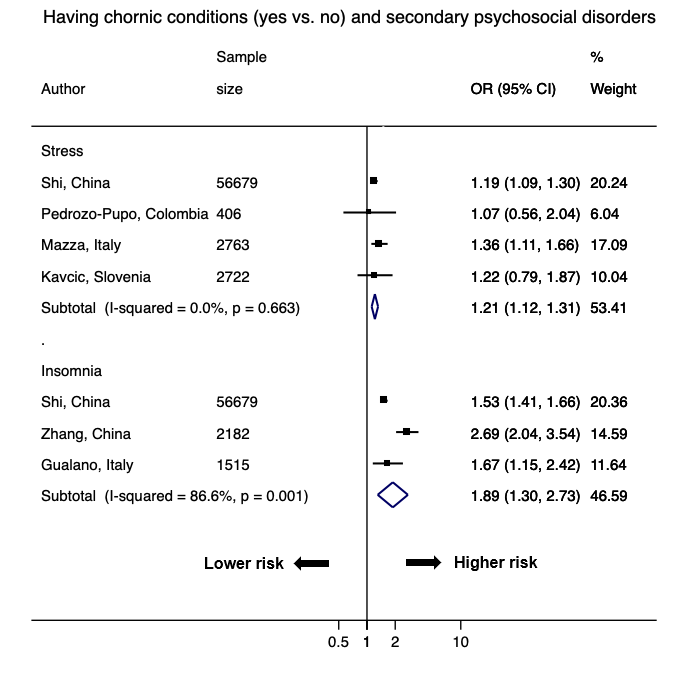


**
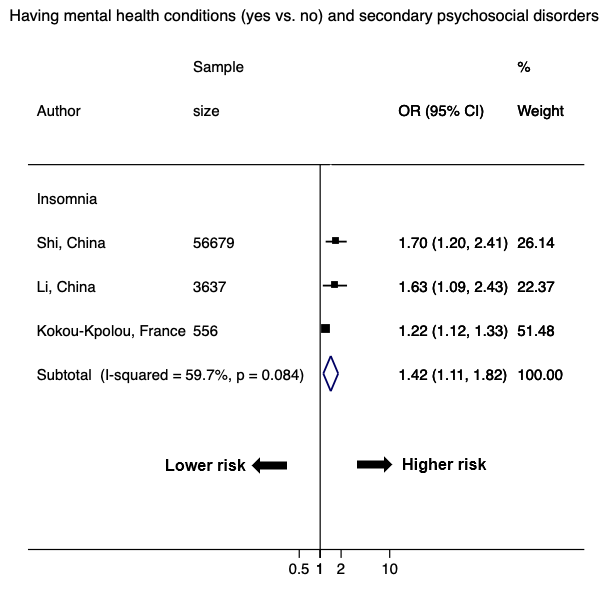
**

**C)**

**Legend:** The size of the data markers indicates the weight of the study, which is the inverse variance of the effect estimate. The diamond data markers indicate the pooled ORs.

**Abbreviations:** PTSD/PTSS, post-traumatic stress disorder/symptoms.

**S6 Fig. Forest plot of media exposure (longer vs. shorter), social support (yes vs. no), physical activity (longer vs. shorter) and anxiety and depression.**

**
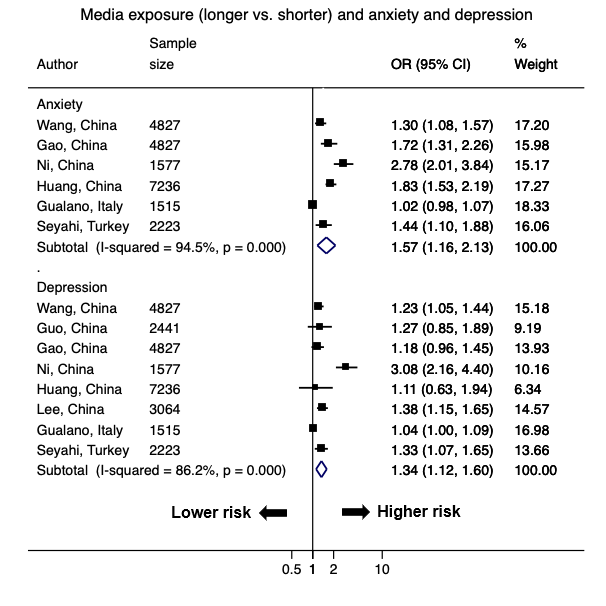
**

**A)**

**
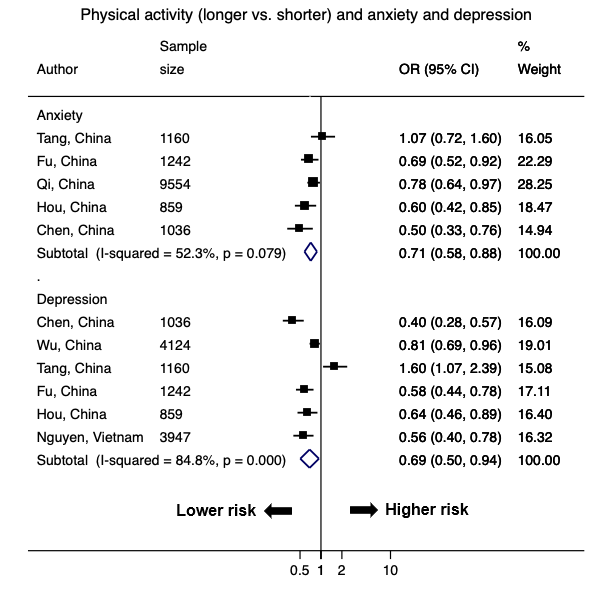

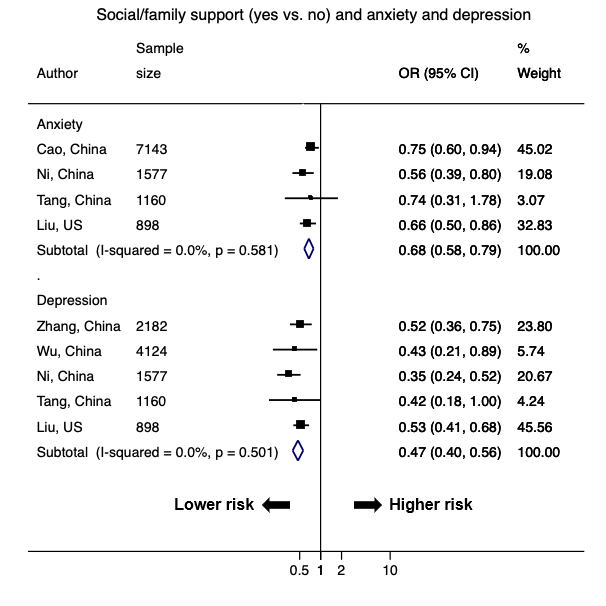
**

**C)**

**B)**

**Legend:** The size of the data markers indicates the weight of the study, which is the inverse variance of the effect estimate. The diamond data markers indicate the pooled ORs.

**S7 Fig. Forest plot of the association between media exposure (longer vs. shorter) and secondary outcomes of psychological distress.**


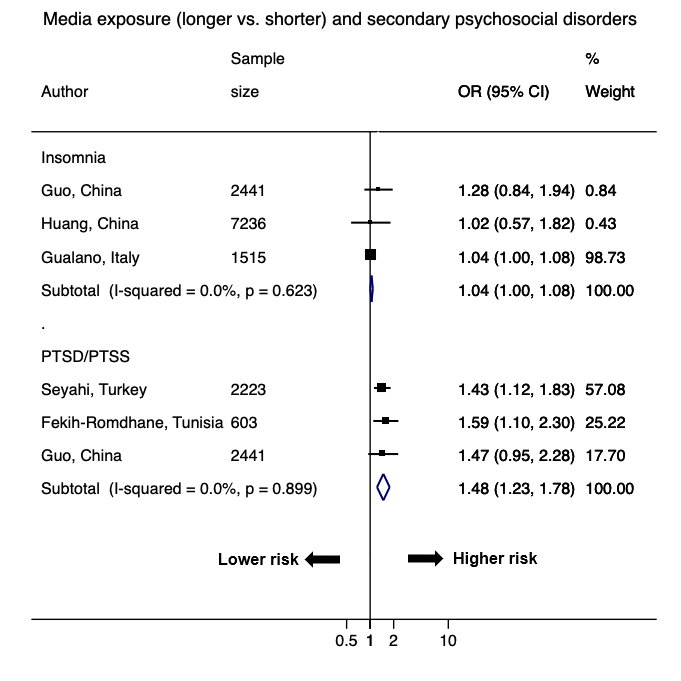


**Legend:** The size of the data markers indicates the weight of the study, which is the inverse variance of the effect estimate. The diamond data markers indicate the pooled ORs.

**Abbreviations:** PTSD/PTSS, post-traumatic stress disorder/symptoms.

**
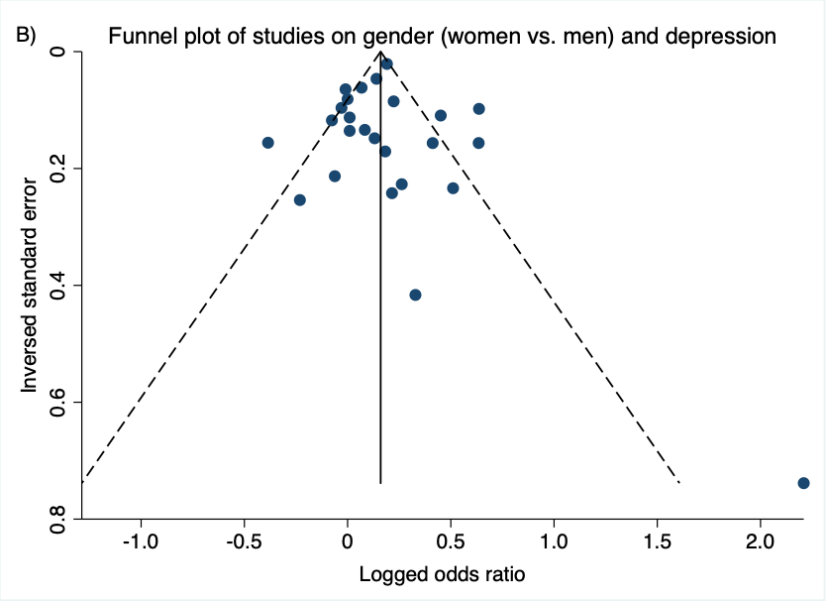

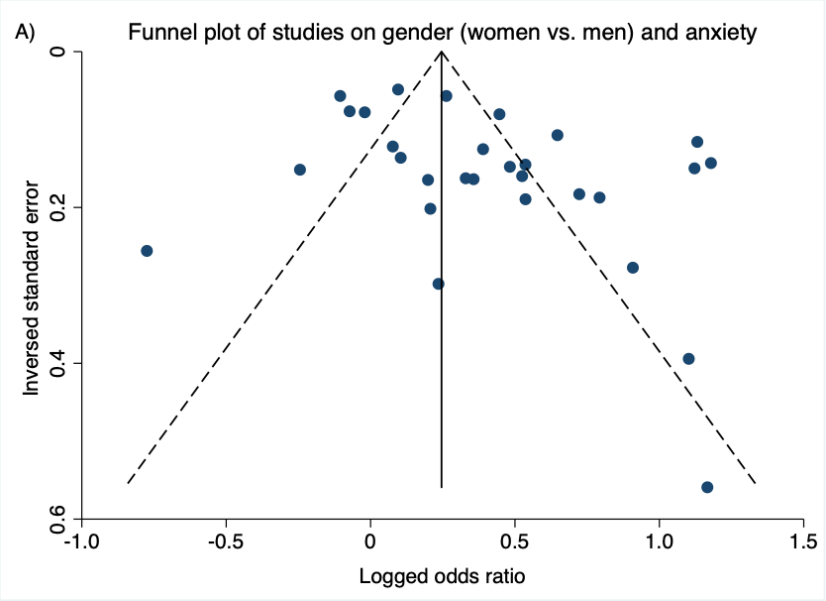
S8 Fig. Potential publication bias tested by funnel plots on gender, age, and socioeconomic status (education, income) with anxiety and depression.**

**
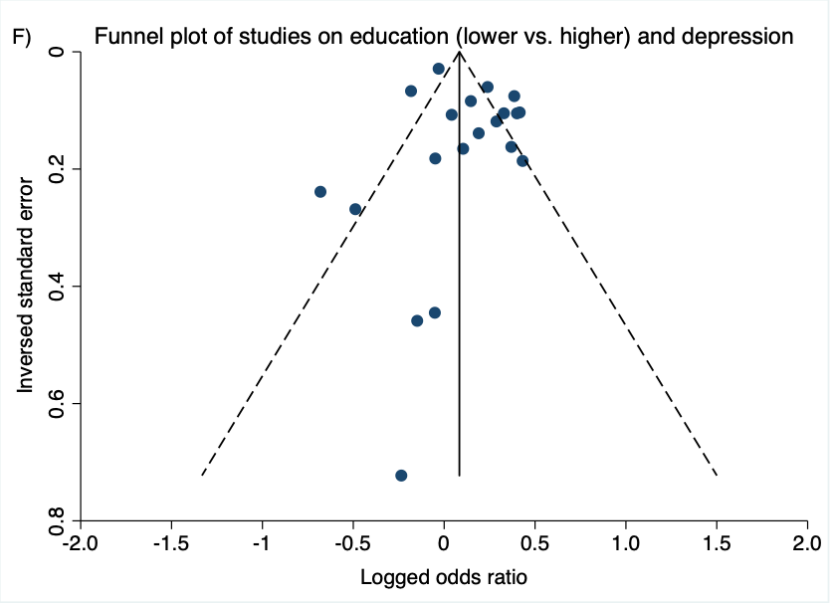

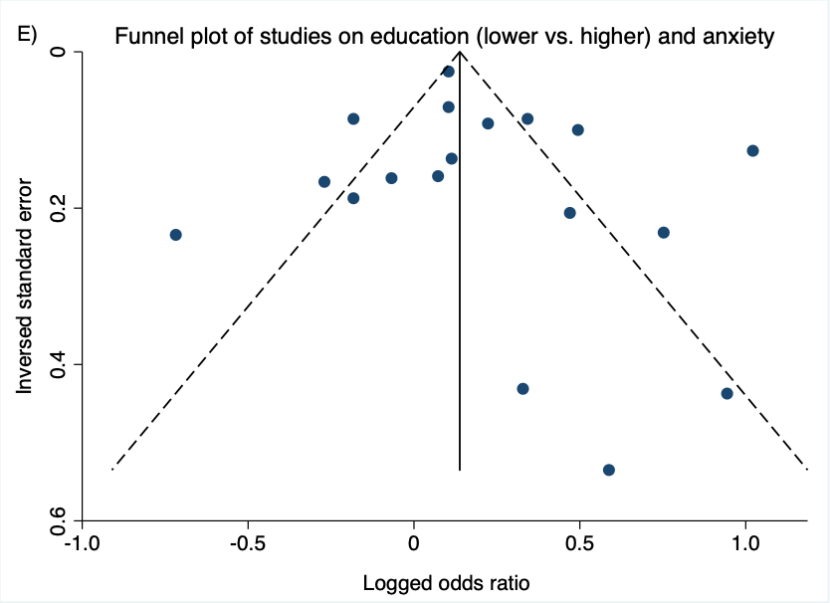

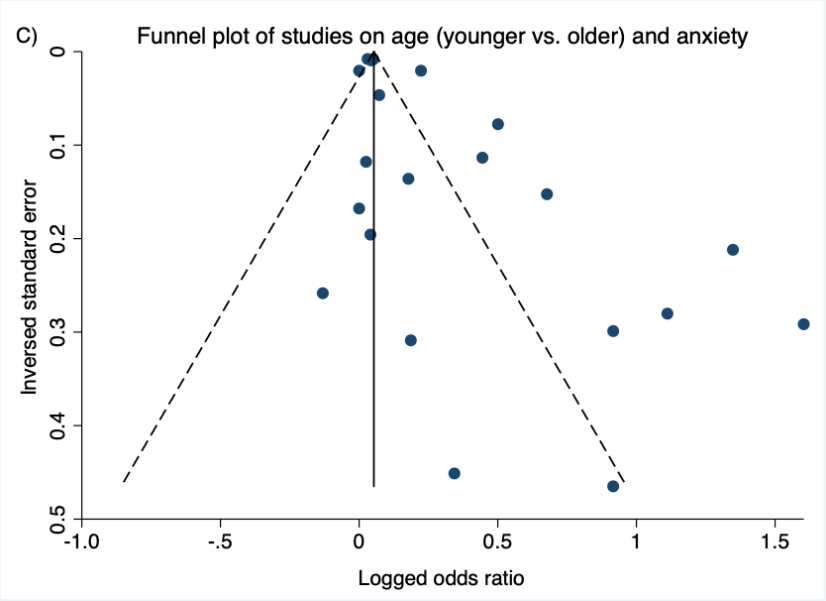

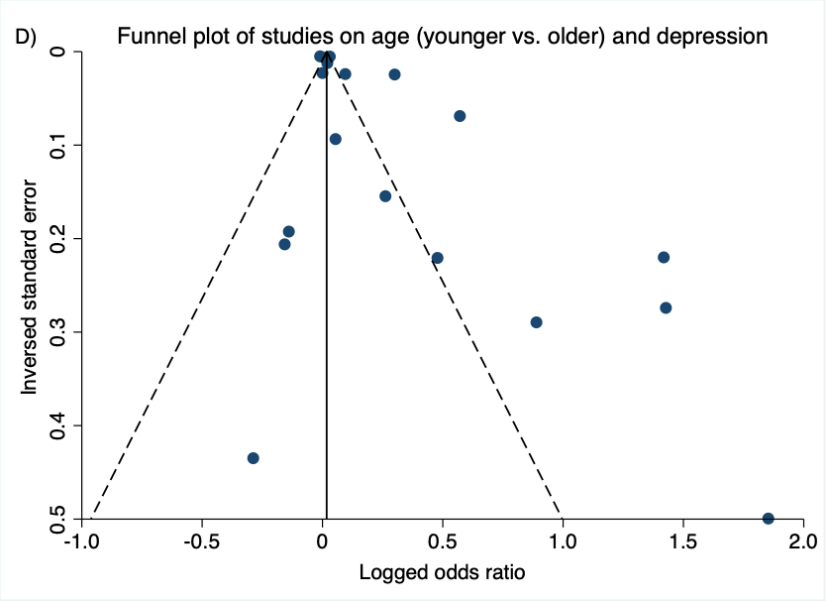
**

**
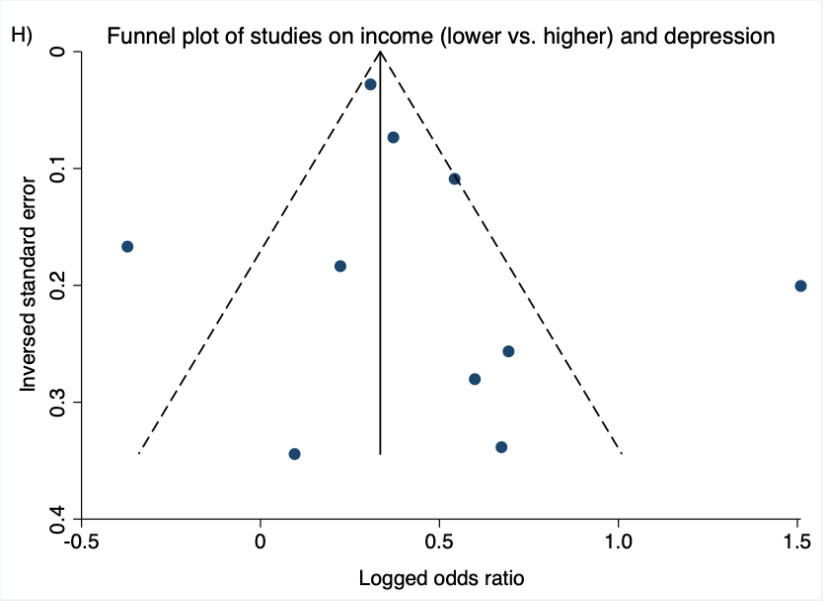

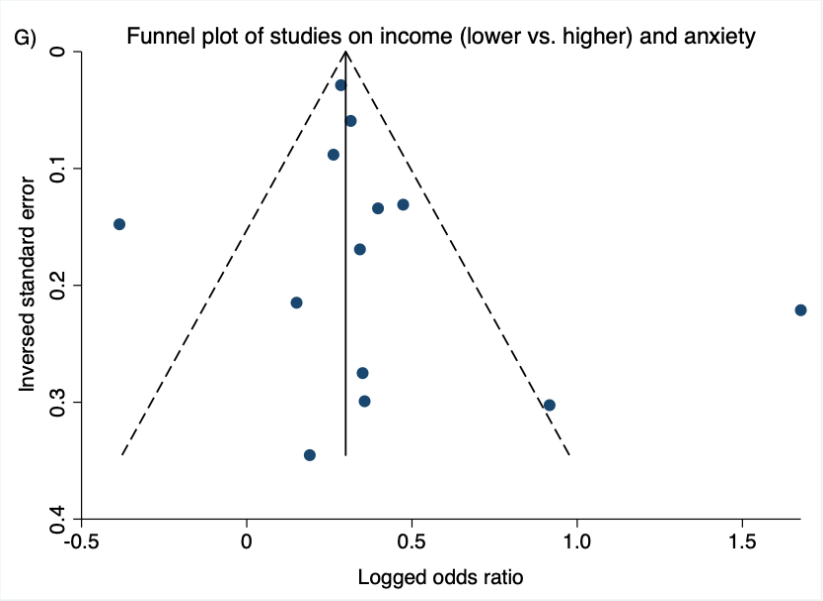
**

**Legend:** The dashed line is the pseudo 95% confidence intervals produced by the funnel plots. It corresponds to the 95% confidence interval for a given standard error. Egger’s test was significant for C) age and anxiety and D) age and depression (*P* ≤0.01). Egger’s test was not significant for other factors with anxiety and depression.

**S1 Table.** **Assessment of study quality using Joanna Briggs Institute scores for studies included in the meta-analysis.**

| **Author, study location** | **Participants and setting described in detail, including similarity of controls** | **Criteria for inclusion**  **clearly defined and exposures similarly measured** | **Exposure measured in valid and reliable way^a^** | **Objective, standard criteria used for measurement of condition** | **Confounding factors identified** | **Strategies to deal with confounding**  **factors stated** | **Outcomes**  **measured**  **in valid and**  **reliable way** | **Appropriate**  **statistical**  **analysis**  **used?** | **Total Joanna Briggs Institute scores** |
| --- | --- | --- | --- | --- | --- | --- | --- | --- | --- |
| Mazza, Italy [1] | **+** | **+** | **+** | **+** | **+** | **+** | **+** | **+** | **8** |
| Moccia, Italy [2] | **+** | **+** | **+** | **+** | **+** | **+** | **+** | **+** | **8** |
| Li, China [3] | **+** | **+** | **+** | **+** | **-** | **-** | **+** | **+** | **6** |
| Li, China [4] | **+** | **+** | **+** | **+** | **+** | **+** | **+** | **+** | **8** |
| Huang, China [5] | **+** | **+** | **+** | **+** | **+** | **+** | **+** | **+** | **8** |
| Li, China [6] | **+** | **-** | **+** | **+** | **-** | **+** | **+** | **+** | **6** |
| Özdin, Turkey [7] | **+** | **-** | **+** | **+** | **-** | **+** | **+** | **+** | **6** |
| Zhang, China [8] | **+** | **+** | **+** | **+** | **+** | **+** | **+** | **+** | **8** |
| Gao, China [9] | **+** | **+** | **+** | **+** | **+** | **+** | **+** | **+** | **8** |
| Xie, China [10] | **+** | **+** | **+** | **+** | **-** | **+** | **+** | **+** | **7** |
| Chang, China [11] | **+** | **+** | **+** | **+** | **+** | **+** | **+** | **+** | **8** |
| Ni, China [12] | **+** | **+** | **+** | **+** | **-** | **+** | **+** | **+** | **7** |
| Nguyen, Vietnam [13] | **+** | **+** | **+** | **+** | **+** | **+** | **+** | **+** | **8** |
| Zhou, China [14] | **+** | **+** | **+** | **+** | **+** | **+** | **+** | **+** | **8** |
| Iasevoli, Italy [15] | **+** | **+** | **+** | **+** | **-** | **+** | **+** | **+** | **7** |
| Hao, China [16] | **+** | **+** | **+** | **+** | **+** | **+** | **+** | **+** | **8** |
| Wang, China [17] | **+** | **+** | **-** | **+** | **+** | **+** | **+** | **+** | **7** |
| Cao, China [18] | **+** | **+** | **+** | **+** | **+** | **+** | **+** | **+** | **8** |
| Chen, China [19] | **+** | **-** | **+** | **+** | **+** | **+** | **+** | **+** | **7** |
| Guo, China [20] | **+** | **+** | **+** | **+** | **+** | **+** | **+** | **+** | **8** |
| Smith, UK [21] | **+** | **+** | **+** | **+** | **+** | **+** | **+** | **+** | **8** |
| Liu, US [22] | **+** | **+** | **+** | **+** | **+** | **+** | **+** | **+** | **8** |
| Costantini, Italy [23] | **+** | **-** | **+** | **+** | **-** | **+** | **+** | **+** | **6** |
| Pedrozo-Pupo, Colombia [24] | **+** | **+** | **+** | **+** | **-** | **+** | **+** | **+** | **7** |
| Chen, China [25] | **+** | **+** | **+** | **+** | **+** | **+** | **+** | **+** | **8** |
| Gómez-Salgado, Spain [26] | **+** | **+** | **+** | **+** | **+** | **+** | **+** | **+** | **8** |
| Forte, Italy [27] | **+** | **+** | **+** | **+** | **-** | **+** | **+** | **+** | **7** |
| Wong, Iran [28] | **+** | **+** | **+** | **+** | **+** | **+** | **+** | **+** | **8** |
| Preis, US [29] | **+** | **+** | **+** | **+** | **+** | **+** | **+** | **+** | **8** |
| Wu, China [30] | **+** | **+** | **+** | **+** | **+** | **+** | **+** | **+** | **8** |
| de Bruin, US [31] | **+** | **+** | **+** | **+** | **+** | **+** | **+** | **+** | **8** |
| Kavčič, Slovenia [32] | **+** | **-** | **+** | **+** | **+** | **+** | **+** | **+** | **7** |
| Zhou, China [33] | **+** | **+** | **+** | **+** | **-** | **+** | **+** | **+** | **7** |
| Zhu, China [34] | **+** | **-** | **-** | **+** | **+** | **+** | **+** | **+** | **6** |
| Wang, China [35] | **+** | **+** | **+** | **+** | **-** | **+** | **+** | **+** | **7** |
| Qi, China [36] | **+** | **+** | **+** | **+** | **+** | **+** | **+** | **+** | **8** |
| Wang, China [37] | **+** | **+** | **+** | **+** | **-** | **+** | **+** | **+** | **7** |
| Zhou, China [38] | **+** | **-** | **+** | **+** | **-** | **+** | **+** | **+** | **6** |
| Tang, China [39] | **+** | **+** | **+** | **+** | **+** | **+** | **+** | **+** | **8** |
| Verma, India [40] | **+** | **+** | **-** | **+** | **-** | **+** | **+** | **+** | **6** |
| Gualano, Italy [41] | **+** | **+** | **+** | **+** | **+** | **+** | **+** | **+** | **8** |
| Kokou-Kpolou, France [42] | **+** | **-** | **+** | **+** | **-** | **+** | **+** | **+** | **6** |
| Mechili, Albania [43] | **+** | **-** | **+** | **+** | **+** | **+** | **+** | **+** | **7** |
| Lin, China [44] | **+** | **+** | **+** | **+** | **+** | **+** | **+** | **+** | **8** |
| Ueda, Japan [45] | **+** | **-** | **+** | **+** | **+** | **+** | **+** | **+** | **7** |
| Naser, Jordan [46] | **+** | **+** | **+** | **+** | **-** | **+** | **+** | **+** | **7** |
| Li, UK [47] | **+** | **-** | **+** | **+** | **+** | **+** | **+** | **+** | **7** |
| Fekih-Romdhane, Tunisia [48] | **+** | **+** | **+** | **+** | **-** | **+** | **+** | **+** | **7** |
| Shi, China [49] | **+** | **+** | **+** | **+** | **+** | **+** | **+** | **+** | **8** |
| Qi, China [50] | **+** | **+** | **+** | **+** | **+** | **+** | **+** | **+** | **8** |
| Peng, China [51] | **+** | **+** | **+** | **+** | **+** | **+** | **+** | **+** | **8** |
| Fu, China [52] | **+** | **+** | **+** | **+** | **+** | **+** | **+** | **+** | **8** |
| Palgi, Israel [53] | **+** | **-** | **+** | **+** | **+** | **+** | **+** | **+** | **7** |
| Seyahi, Turkey [54] | **+** | **+** | **+** | **+** | **+** | **+** | **+** | **+** | **8** |
| Lee, China [55] | **+** | **-** | **+** | **+** | **+** | **+** | **+** | **+** | **7** |
| Duan, China [56] | **+** | **+** | **+** | **+** | **-** | **+** | **+** | **+** | **7** |
| Karatzias, Ireland [57] | **+** | **+** | **+** | **+** | **+** | **+** | **+** | **+** | **8** |
| Liu, China [58] | **+** | **+** | **+** | **+** | **+** | **+** | **+** | **+** | **8** |
| Yang, China [59] | **+** | **+** | **+** | **+** | **+** | **+** | **+** | **+** | **8** |
| Hou, China [60] | **+** | **+** | **-** | **+** | **-** | **+** | **+** | **+** | **6** |
| Wang, China [61] | **+** | **+** | **+** | **+** | **+** | **+** | **+** | **+** | **8** |
| Ma, China [62] | **+** | **+** | **+** | **+** | **+** | **+** | **+** | **+** | **8** |
| Domínguez-Salas, Spain [63] | **+** | **+** | **+** | **+** | **+** | **+** | **+** | **+** | **8** |
| Huang, China [64] | **+** | **+** | **+** | **+** | **+** | **+** | **+** | **+** | **8** |
| Liu, China [65] | **+** | **+** | **+** | **+** | **+** | **+** | **+** | **+** | **8** |
| Ramasubramanian, India [66] | **+** | **-** | **+** | **+** | **-** | **+** | **+** | **+** | **6** |
| Ben-Ezra, China [67] | **+** | **-** | **+** | **+** | **+** | **+** | **+** | **+** | **7** |
| Mosli, Saudi Arabia [68] | **+** | **+** | **+** | **+** | **+** | **+** | **+** | **+** | **8** |

^a^Defined as if the survey was done during COVID-19 pandemic and questions were asked about exposure to COVID-19 virus.

**S2 Table. Stratified analyses of factors and risks of anxiety and depression by study locations.**

|  | **Anxiety** | | | |  | **Depression** | | | |  |
| --- | --- | --- | --- | --- | --- | --- | --- | --- | --- | --- |
|  | **OR (95% CI)^a^** | ***N* of studies** | ***I*^2^ (%)** | **P for heterogeneity** | **P for meta-regression** | **RR (95% CI)^a^** | ***N* of studies** | ***I*^2^ (%)** | **P for heterogeneity** | **P for meta-regression** |
| **Gender (women vs. men)** | |  |  |  |  |  |  |  |  |  |
| Overall results | 1.48 (1.29-1.71) | 29 | 90.8% | <0.001 |  | 1.16 (1.07-1.26) | 25 | 75.0% | <0.001 |  |
| Stratified by locations |  |  |  |  | 0.037 |  |  |  |  | 0.98 |
| Asia | 1.26 (1.01-1.57) | 11 | 90.6% | <0.001 |  | 1.11 (0.96-1.29) | 15 | 84.6% | <0.001 |  |
| Middle East | - | 2 | - | - |  | - | 2 | - | - |  |
| Europe | 1.12 (0.93-1.34) | 4 | 15.4% | 0.14 |  | - | 2 | - | - |  |
| U.S. | - | 1 | - | - |  | - | 0 | - | - |  |
| **Age (younger vs. older)** | |  |  |  |  |  |  |  |  |  |
| Overall results | 1.20 (1.13-1.26) | 21 | 91.7% | <0.001 |  | 1.13 (1.08-1.18) | 18 | 95.1% | <0.001 |  |
| Stratified by locations |  |  |  |  | 0.87 |  |  |  |  | 0.40 |
| Asia | 1.36 (1.19-1.56) | 13 | 91.3% | <0.001 |  | 1.25 (1.12-1.40) | 13 | 95.9% | <0.001 |  |
| Middle East | 1.23 (0.86-1.76) | 3 | 76.7% | - |  | - | 2 | - | - |  |
| Europe | 1.10 (1.03-1.18) | 4 | 93.3% | <0.001 |  | 1.02 (0.99-1.06) | 3 | 89.8% | <0.001 |  |
| U.S. | - | 0 | - | - |  | - | 0 | - | - |  |
| **Education (lower vs. higher)** | |  |  |  |  |  |  |  |  |  |
| Overall results | 1.21 (1.05-1.40) | 18 | 86.1% | <0.001 |  | 1.15 (1.03-1.29) | 20 | 82.0% | 0.016 |  |
| Stratified by locations |  |  |  |  | 0.81 |  |  |  |  | 0.27 |
| Asia | 1.26 (1.01-1.57) | 11 | 90.6% | <0.001 |  | 1.11 (0.96-1.29) | 15 | 84.6% | <0.001 |  |
| Middle East | - | 2 | - | - |  | - | 2 | - | - |  |
| Europe | 1.12 (0.93-1.34) | 4 | 45.4% | 0.14 |  | - | 2 | - | - |  |
| U.S. | - | 1 | - | - |  | - | 1 | - | - |  |
| **Income (lower vs. higher)** | |  |  |  |  |  |  |  |  |  |
| Overall results | 1.45 (1.24-1.69) | 13 | 82.3% | <0.001 |  | 1.56 (1.26-1.92) | 10 | 85.4% | <0.001 |  |
| Stratified by locations |  |  |  |  | 0.69 |  |  |  |  | 0.62 |
| Asia | 1.46 (1.20-1.78) | 9 | 87.3% | <0.001 |  | 1.47 (1.12-1.91) | 7 | 89.0% | <0.001 |  |
| Middle East | - | 1 | - | - |  | - | 1 | - | - |  |
| Europe | - | 1 | - | - |  | - | 1 | - | - |  |
| U.S. | - | 2 | - | - |  | - | 1 | - | - |  |
| **Current employment (yes vs. no)** | |  |  |  |  |  |  |  |  |  |
| Overall results | 0.89 (0.78-1.02) | 8 | 26.6% | 0.22 |  | 0.76 (0.61-0.95) | 7 | 63.8% | 0.011 |  |
| Stratified by locations |  |  |  |  | 0.31 |  |  |  |  | 0.42 |
| Asia | 0.87 (0.76-1.00) | 4 | 0% | 0.48 |  | 0.95 (0.61-1.49) | 3 | 80% | 0.007 |  |
| Middle East | - | 2 | - | - |  | - | 2 | - | - |  |
| Europe | - | 1 | - | - |  | - | 1 | - | - |  |
| U.S. | - | 1 | - | - |  | - | 1 | - | - |  |
| **Residential area (rural vs. urban)** | |  |  |  |  |  |  |  |  |  |
| Overall results | 1.13 (1.00-1.29) | 7 | 82.9% | <0.001 |  | 0.98 (0.85-1.12) | 7 | 81.6% | <0.001 |  |
| Stratified by locations |  |  |  |  | 0.11 |  |  |  |  | 0.15 |
| Asia | 1.11 (0.98-1.25) | 6 | 83.2% | <0.001 |  | 0.95 (0.83-1.08) | 6 | 81.9% | <0.001 |  |
| Middle East | - | 0 | - | - |  | - | 0 | - | - |  |
| Europe | - | 1 | - | - |  | - | 1 | - | - |  |
| U.S. | - | 0 | - | - |  | - | 0 | - | - |  |
| **Suspected/confirmed COVID-19 cases (yes vs. no)** | | |  |  |  |  |  |  |  |  |
| Overall results | 1.70 (1.41-2.06) | 15 | 79.5% | <0.001 |  | 1.84 (1.39-2.43) | 8 | 73.5% | <0.001 |  |
| Stratified by locations |  |  |  |  | 0.66 |  |  |  |  | 0.58 |
| Asia | 1.91 (1.50-2.42) | 10 | 60.7% | 0.006 |  | 1.98 (1.47-2.67) | 6 | 57.9% | 0.036 |  |
| Middle East | - | 2 | - | - |  | - | 1 | - | - |  |
| Europe | - | 2 | - | - |  | - | 1 | - | - |  |
| U.S. | - | 1 | - | - |  | - | 0 | - | - |  |
| **Living in the hard-hit area (yes vs. no)** | |  |  |  |  |  |  |  |  |  |
| Overall results | 1.57 (1.36-1.81) | 10 | 73.9% | <0.001 |  | 1.33 (1.16-1.53) | 10 | 69.1% | 0.001 |  |
| Stratified by locations |  |  |  |  | 0.47 |  |  |  |  | 0.68 |
| Asia | 1.61 (1.38-1.88) | 9 | 73.0% | <0.001 |  | 1.31 (1.11-1.54) | 9 | 72.4% | <0.001 |  |
| Middle East | - | 0 | - | - |  | - | 0 | - | - |  |
| Europe | - | 0 | - | - |  | - | 0 | - | - |  |
| U.S. | - | 1 | - | - |  | - | 1 | - | - |  |
| **Pre-existing physical conditions (yes vs. no)** | |  |  |  |  |  |  |  |  |  |
| Overall results | 1.48 (1.21-1.81) | 11 | 65.2% | 0.001 |  | 1.42 (1.12-1.80) | 11 | 89.0% | <0.001 |  |
| Stratified by locations |  |  |  |  | 0.09 |  |  |  |  | 0.11 |
| Asia | - | 2 | - | - |  | 1.66 (1.12-2.45) | 5 | 93.7% | <0.001 |  |
| Middle East | 0.98 (0.63-1.53) | 3 | 29.9% | 0.24 |  | - | 2 | - | - |  |
| Europe | 1.60 (1.19-2.14) | 5 | 68.3% | 0.013 |  | 1.44 (1.04-1.98) | 4 | 75.4% | 0.007 |  |
| U.S. | - | 1 | - | - |  | - | 0 | - | - |  |
| **Pre-existing mental conditions (yes vs. no)** | |  |  |  |  |  |  |  |  |  |
| Overall results | 1.82 (1.34-2.48) | 6 | 70.8% | 0.004 |  | 1.75 (0.98-3.14) | 6 | 93.5% | <0.001 |  |
| Stratified by locations |  |  |  |  | 0.13 |  |  |  |  | 0.38 |
| Asia | - | 2 | - | - |  | - | 2 | - | - |  |
| Middle East | 1.57 (0.90-2.73) | 3 | 68.0% | 0.044 |  | 1.34 (0.75-2.41) | 3 | 83.1% | 0.003 |  |
| Europe | - | 0 | - | - |  | - | 0 | - | - |  |
| U.S. | - | 1 | - | - |  | - | 1 | - | - |  |
| **Media exposure (longer vs. shorter)** | |  |  |  |  |  |  |  |  |  |
| Overall results | 1.57 (1.16-2.13) | 6 | 94.5% | <0.001 |  | 1.34 (1.12-1.60) | 8 | 86.2% | <0.001 |  |
| Stratified by locations |  |  |  |  | 0.17 |  |  |  |  | 0.47 |
| Asia | 1.80 (1.36-2.38) | 4 | 82.9% | 0.001 |  | 1.43 (1.13-1.80) | 6 | 79.4% | <0.001 |  |
| Middle East | - | 0 | - | - |  | - | 0 | - | - |  |
| Europe | - | 2 | - | - |  | - | 2 | - | - |  |
| U.S. | - | 0 | - | - |  | - | 0 | - | - |  |
| **Social/family support (yes vs. no)** | |  |  |  |  |  |  |  |  |  |
| Overall results | 0.68 (0.58-0.79) | 4 | 0% | 0.58 |  | 0.47 (0.40-0.56) | 5 | 0% | 0.50 |  |
| Stratified by locations |  |  |  |  | 0.89 |  |  |  |  | 0.42 |
| Asia | 0.69 (0.57-0.83) | 3 | 0% | 0.54 |  | 0.43 (0.34-0.55) | 4 | 0% | 0.54 |  |
| Middle East | - | 0 | - | - |  | - | 0 | - | - |  |
| Europe | - | 0 | - | - |  | - | 0 | - | - |  |
| U.S. | - | 1 | - | - |  | - | 1 | - | - |  |
| **Physical activity (longer vs. shorter)** | |  |  |  |  |  |  |  |  |  |
| Overall results | 0.71 (0.58-0.88) | 5 | 52.3% | 0.08 |  | 0.69 (0.50-0.94) | 6 | 84.8% | <0.001 |  |
| Stratified by locations |  |  |  |  | - |  |  |  |  | - |
| Asia | 0.71 (0.58-0.88) | 5 | 52.3% | 0.08 |  | 0.69 (0.50-0.94) | 6 | 84.8% | <0.001 |  |
| Middle East | - | 0 | - | - |  | - | 0 | - | - |  |
| Europe | - | 0 | - | - |  | - | 0 | - | - |  |
| U.S. | - | 0 | - | - |  | - | 0 | - | - |  |

**Abbreviations:** OR, odds ratio; CI, confidence interval.

^a^The odds ratios were calculated using the random-effects model among studies with three or more data points.

**S3 Table. Sensitivity analyses of factors and risks of anxiety and depression among studies using the same instrument and cut-off values.**

|  | **Anxiety** | | | |  |  | **Depression** | | | |  |
| --- | --- | --- | --- | --- | --- | --- | --- | --- | --- | --- | --- |
|  | **OR**  **(95% CI)^a^** | ***N* of studies** | ***I*^2^ (%)** | **P for heterogeneity** | **P for meta-regression** |  | **RR**  **(95% CI)^a^** | ***N* of studies** | ***I*^2^ (%)** | **P for heterogeneity** | **P for meta-regression** |
| **Gender (women vs. men)** | |  |  |  | 0.66 | **Age (younger vs. older)** | |  |  |  | - |
| Overall results | 1.20  (1.13-1.26) | 21 | 91.7% | <0.001 |  | Overall results | 1.13  (1.08-1.18) | 18 | 95.1% | <0.001 |  |
| Use GAD-7 ≥10 | 1.13  (0.93-1.38) | 6 | 68.6% | 0.007 |  | Use PHQ-9 ≥10 | 1.06  (0.98-1.16) | 5 | 92.1% | <0.001 |  |
| Use GAD-7 ≥5 | 1.33  (0.91-1.93) | 5 | 69.6% | <0.001 |  |  |  |  |  |  |  |
| Use GAD-2 ≥3 | 1.55  (1.07-2.24) | 3 | 76.2% | 0.015 |  |  |  |  |  |  |  |
| **Age (younger vs. older)** | |  |  |  | **-** | **Gender (women vs. men)** | |  |  |  | - |
| Overall results | 1.48  (1.29-1.71) | 29 | 90.8% | <0.001 |  | Overall results | 1.16  (1.07-1.26) | 25 | 75.0% | <0.001 |  |
| Use GAD-7 ≥10 | 1.17  (1.03-1.34) | 10 | 76.4% | <0.001 |  | Use PHQ-9 ≥10 | 1.13  (0.93-1.38) | 6 | 68.6% | 0.007 |  |
| **Education (lower vs. higher)** | |  |  |  | **-** | **Education (lower vs. higher)** | |  |  |  | - |
| Overall results | 1.21  (1.05-1.40) | 18 | 86.1% | <0.001 |  | Overall results | 1.15  (1.03-1.29) | 20 | 82.0% | 0.016 |  |
| Use GAD-7 ≥10 | 1.51  (0.98-2.35) | 5 | 91.4% | <0.001 |  | Use PHQ-9 ≥10 | 1.08  (0.80-1.47) | 5 | 82.5% | <0.001 |  |
| **Income (lower vs. higher)** | |  |  |  | - |  |  |  |  |  |  |
| Overall results | 1.45  (1.24-1.69) | 13 | 82.3% | <0.001 |  |  |  |  |  |  |  |
| Use GAD-7 ≥5 | 1.65  (0.75-3.62) | 3 | 96.7% | <0.001 |  |  |  |  |  |  |  |
| **Living in the hard-hit area (yes vs. no)** | | |  |  | - |  |  |  |  |  |  |
| Overall results | 1.57  (1.36-1.81) | 10 | 73.9% | <0.001 |  |  |  |  |  |  |  |
| Use GAD-7 ≥10 | 1.68  (1.16-2.43) | 4 | 77.9% | 0.004 |  |  |  |  |  |  |  |
| **Suspected/confirmed COVID-19 cases (yes vs. no)** | | | |  | - |  |  |  |  |  |  |
| Overall results | 1.70  (1.41-2.06) | 15 | 79.5% | <0.001 |  |  |  |  |  |  |  |
| Use GAD-2 ≥3 | 1.75  (1.15-2.66) | 3 | 67.3% | 0.047 |  |  |  |  |  |  |  |
| **Pre-existing physical conditions (yes vs. no)** | | | |  | - |  |  |  |  |  |  |
| Overall results | 1.48  (1.21-1.81) | 11 | 65.2% | 0.001 |  |  |  |  |  |  |  |
| Use GAD-7 ≥10 | 1.26  (0.79-2.00) | 3 | 73.9% | 0.022 |  |  |  |  |  |  |  |
| **Physical activity (longer vs. shorter)** | | |  |  | - |  |  |  |  |  |  |
| Overall results | 0.71  (0.58-0.88) | 5 | 52.3% | 0.08 |  |  |  |  |  |  |  |
| Use GAD-7 ≥5 | 0.80  (0.65-0.98) | 3 | 34.7% | 0.22 |  |  |  |  |  |  |  |

**Abbreviations:** OR, odds ratio; CI, confidence interval.

^a^The odds ratios were calculated using the random-effects model among studies with three or more data points.

**S4 Table. Sensitivity analyses of factors and risks of anxiety and depression among studies excluding patients and healthcare professionals.**

|  | **Anxiety** | | | | **Depression** | | | |
| --- | --- | --- | --- | --- | --- | --- | --- | --- |
|  | **OR (95% CI)^a^** | ***N* of studies** | ***I*^2^ (%)** | **P for heterogeneity** | **RR (95% CI)^a^** | ***N* of studies** | ***I*^2^ (%)** | **P for heterogeneity** |
| **Gender (women vs. men)** |  |  |  |  |  |  |  |  |
| Overall results | 1.48 (1.29-1.71) | 29 | 90.8% | <0.001 | 1.16 (1.07-1.26) | 25 | 75.0% | <0.001 |
| Studies excluding patients and  healthcare professionals | 1.44 (1.25-1.67) | 26 | 90.6% | <0.001 | 1.12 (1.04-1.22) | 21 | 71.3% | <0.001 |
| **Age (younger vs. older)** |  |  |  |  |  |  |  |  |
| Overall results | 1.20 (1.13-1.26) | 21 | 91.7% | <0.001 | 1.13 (1.08-1.18) | 18 | 95.1% | <0.001 |
| Studies excluding patients and  healthcare professionals | 1.30 (1.21-1.41) | 19 | 92.3% | <0.001 | 1.20 (1.13-1.28) | 16 | 95.5% | <0.001 |
| **Education (lower vs. higher)** |  |  |  |  |  |  |  |  |
| Overall results | 1.21 (1.05-1.40) | 18 | 86.1% | <0.001 | 1.15 (1.03-1.29) | 20 | 82.0% | 0.016 |
| Studies excluding patients and  healthcare professionals | 1.18 (1.02-1.37) | 16 | 87.4% | <0.001 | 1.14 (1.01-1.28) | 17 | 84.3% | <0.001 |
| **Income (lower vs. higher)** |  |  |  |  |  |  |  |  |
| Overall results | 1.45 (1.24-1.69) | 13 | 82.3% | <0.001 | 1.56 (1.26-1.92) | 10 | 85.4% | <0.001 |
| Studies excluding patients and  healthcare professionals | 1.45 (1.24-1.70) | 13 | 82.3% | <0.001 | 1.56 (1.26-1.92) | 10 | 85.4% | <0.001 |
| **Current employment (yes vs. no)** |  |  |  |  |  |  |  |  |
| Overall results | 0.89 (0.78-1.02) | 8 | 26.6% | 0.22 | 0.76 (0.61-0.95) | 7 | 63.8% | 0.011 |
| Studies excluding patients and  healthcare professionals | 0.89 (0.78-1.02) | 8 | 26.6% | 0.22 | 0.76 (0.61-0.95) | 7 | 63.8% | 0.011 |
| **Residential area (rural vs. urban)** |  |  |  |  |  |  |  |  |
| Overall results | 1.13 (1.00-1.29) | 7 | 82.9% | <0.001 | 0.98 (0.85-1.12) | 7 | 81.6% | <0.001 |
| Studies excluding patients and  healthcare professionals | 1.13 (1.00-1.29) | 7 | 82.9% | <0.001 | 0.98 (0.85-1.12) | 7 | 81.6% | <0.001 |
| **Suspected/confirmed COVID-19 cases (yes vs. no)** | |  |  |  |  |  |  |  |
| Overall results | 1.70 (1.41-2.06) | 15 | 79.5% | <0.001 | 1.84 (1.39-2.43) | 8 | 73.5% | <0.001 |
| Studies excluding patients and  healthcare professionals | 1.71 (1.41-2.07) | 14 | 81.0% | <0.001 | 1.79 (1.34-2.38) | 7 | 75.9% | <0.001 |
| **Living in the hard-hit area (yes vs. no)** | |  |  |  |  |  |  |  |
| Overall results | 1.57 (1.36-1.81) | 10 | 73.9% | <0.001 | 1.33 (1.16-1.53) | 10 | 69.1% | 0.001 |
| Studies excluding patients and  healthcare professionals | 1.64 (1.43-1.87) | 9 | 67.0% | 0.002 | 1.32 (1.12-1.54) | 9 | 72.5% | <0.001 |
| **Pre-existing physical conditions (yes vs. no)** | |  |  |  |  |  |  |  |
| Overall results | 1.48 (1.21-1.81) | 11 | 65.2% | 0.001 | 1.42 (1.12-1.80) | 11 | 89.0% | <0.001 |
| Studies excluding patients and  healthcare professionals | 1.47 (1.17-1.85) | 10 | 68.2% | 0.001 | 1.38 (1.06-1.81) | 10 | 89.9% | <0.001 |
| **Pre-existing mental conditions (yes vs. no)** | |  |  |  |  |  |  |  |
| Overall results | 1.82 (1.34-2.48) | 6 | 70.8% | 0.004 | 1.75 (0.98-3.14) | 6 | 93.5% | <0.001 |
| Studies excluding patients and  healthcare professionals | 1.84 (1.30-2.61) | 4 | 78.7% | 0.003 | 1.74 (0.85-3.54) | 4 | 96.0% | <0.001 |
| **Media exposure (longer vs. shorter)** | |  |  |  |  |  |  |  |
| Overall results | 1.57 (1.16-2.13) | 6 | 94.5% | <0.001 | 1.34 (1.12-1.60) | 8 | 86.2% | <0.001 |
| Studies excluding patients and  healthcare professionals | 1.60 (1.14-2.27) | 5 | 95.4% | <0.001 | 1.34 (1.10-1.64) | 7 | 87.3% | <0.001 |
| **Social/family support (yes vs. no)** |  |  |  |  |  |  |  |  |
| Overall results | 0.68 (0.58-0.79) | 4 | 0% | 0.58 | 0.47 (0.40-0.56) | 5 | 0% | 0.50 |
| Studies excluding patients and  healthcare professionals | 0.68 (0.58-0.79) | 4 | 0% | 0.58 | 0.47 (0.40-0.56) | 5 | 0% | 0.50 |
| **Physical activity (longer vs. shorter)** | |  |  |  |  |  |  |  |
| Overall results | 0.71 (0.58-0.88) | 5 | 52.3% | 0.08 | 0.69 (0.50-0.94) | 6 | 84.8% | <0.001 |
| Studies excluding patients and  healthcare professionals | 0.71 (0.58-0.88) | 5 | 52.3% | 0.08 | 0.69 (0.50-0.94) | 6 | 84.8% | <0.001 |

**Abbreviations:** OR, odds ratio; CI, confidence interval.

^a^The odds ratios were calculated using the random-effects model.

**S5 Table.** **The attributable risk of depression due to the COVID-19 pandemic.**

| **Author, study location** | **Study period** | **Prevalence of COVID-19 infection** | **Prevalence of depression among patients with COVID-19** | **Prevalence of depression among participants without COVID-19** | **Attributable risk of depression due to the COVID-19 pandemic^a^** |
| --- | --- | --- | --- | --- | --- |
| Ma, China^62^ | February 24 and March 8, 2020 | 0.56 per 10,000 people^69,70^ | 51.6% | 41.9% | 9.70% |
| Nguyen, Vietnam^13^ | February 14 and March 2, 2020 | 0.0017 per 10,000 people^69,71^ | 13.6% | 7.04% | 9.52% |

^a^The attributable risk of depression due to the COVID-19 pandemic was calculated by the formula $\frac{{R1}/{R0}-1}{R1/R0}$, where ${R1}/{R0}$ is the causal risk ratio that measures the risk under exposure (COVID-19).^72^

**REFERENCES**

1. Mazza C, Ricci E, Biondi S, et al. A Nationwide Survey of Psychological Distress among Italian People during the COVID-19 Pandemic: Immediate Psychological Responses and Associated Factors. Int J Environ Res Public Health. 2020;17:3165.

2. Moccia L, Janiri D, Pepe M, et al. Affective temperament, attachment style, and the psychological impact of the COVID-19 outbreak: an early report on the Italian general population. Brain Behav Immun 2020;87:75-9.

3. Li J, Yang Z, Qiu H, et al. Anxiety and depression among general population in China at the peak of the COVID-19 epidemic. World Psychiatry 2020;19:249-50.

4. Li X, Dai T, Wang H, et al. [Clinical analysis of suspected COVID-19 patients with anxiety and depression]. Zhejiang Da Xue Xue Bao Yi Xue Ban 2020;49:203-8.

5. Huang Y, Zhao N. Generalized anxiety disorder, depressive symptoms and sleep quality during COVID-19 outbreak in China: a web-based cross-sectional survey. Psychiatry Res 2020;288:112954.

6. Li Y, Qin Q, Sun Q, Sanford LD, Vgontzas AN, Tang X. Insomnia and psychological reactions during the COVID-19 outbreak in China. J Clin Sleep Med 2020; published online Apr 30. Doi: 10.5664/jcsm.8524.

7. Özdin S, Bayrak Özdin Ş. Levels and predictors of anxiety, depression and health anxiety during COVID-19 pandemic in Turkish society: The importance of gender. Int J Soc Psychiatry 2020; published online May 8. Doi: 10.1177/0020764020927051.

8. Zhang WR, Wang K, Yin L, et al. Mental Health and Psychosocial Problems of Medical Health Workers during the COVID-19 Epidemic in China. Psychother Psychosom 2020;89:242-50.

9. Gao J, Zheng P, Jia Y, et al. Mental health problems and social media exposure during COVID-19 outbreak. PLoS One 2020;15:e0231924.

10. Xie X, Xue Q, Zhou Y, et al. Mental Health Status Among Children in Home Confinement During the Coronavirus Disease 2019 Outbreak in Hubei Province, China. JAMA Pediatr 2020; published online Apr 24. Doi: 10.1001/jamapediatrics.2020.1619.

11. Chang J, Yuan Y, Wang D. [Mental health status and its influencing factors among college students during the epidemic of COVID-19]. Nan Fang Yi Ke Da Xue Xue Bao 2020;40:171-6.

12. Ni MY, Yang L, Leung CMC, et al. Mental Health, Risk Factors, and Social Media Use During the COVID-19 Epidemic and Cordon Sanitaire Among the Community and Health Professionals in Wuhan, China: Cross-Sectional Survey. JMIR Ment Health 2020;7:e19009.

13. Nguyen HC, Nguyen MH, Do BN, et al. People with Suspected COVID-19 Symptoms Were More Likely Depressed and Had Lower Health-Related Quality of Life: The Potential Benefit of Health Literacy. J Clin Med 2020;9:965.

14. Zhou SJ, Zhang LG, Wang LL, et al. Prevalence and socio-demographic correlates of psychological health problems in Chinese adolescents during the outbreak of COVID-19. Eur Child Adolesc Psychiatry 2020;29:749-58.

15. Iasevoli F, Fornaro M, D'Urso G, et al. Psychological distress in patients with serious mental illness during the COVID-19 outbreak and one-month mass quarantine in Italy. Psychol Med 2020; published online May 19. Doi: 10.1017/S0033291720001841.

16. Hao X, Zhou D, Li Z, et al. Severe psychological distress among patients with epilepsy during the COVID-19 outbreak in southwest China. Epilepsia 2020;61:1166-73.

17. Wang Y, Di Y, Ye J, Wei W. Study on the public psychological states and its related factors during the outbreak of coronavirus disease 2019 (COVID-19) in some regions of China. Psychol Health Med 2020; published online Mar 30. Doi: 10.1080/13548506.2020.1746817.

18. Cao W, Fang Z, Hou G, et al. The psychological impact of the COVID-19 epidemic on college students in China. Psychiatry Res 2020;287:112934.

19. Chen F, Zheng D, Liu J, Gong Y, Guan Z, Lou D. Depression and anxiety among adolescents during COVID-19: A cross-sectional study. Brain Behav Immun 2020; published online May 25. Doi: 10.1016/j.bbi.2020.05.061.

20. Guo J, Feng XL, Wang XH, van IMH. Coping with COVID-19: Exposure to COVID-19 and Negative Impact on Livelihood Predict Elevated Mental Health Problems in Chinese Adults. Int J Environ Res Public Health 2020;17: 3857.

21. Smith L, Jacob L, Yakkundi A, et al. Correlates of symptoms of anxiety and depression and mental wellbeing associated with COVID-19: a cross-sectional study of UK-based respondents. Psychiatry Res 2020;291:113138.

22. Liu CH, Zhang E, Wong GTF, Hyun S, Hahm HC. Factors associated with depression, anxiety, and PTSD symptomatology during the COVID-19 pandemic: Clinical implications for U.S. young adult mental health. Psychiatry Res 2020;290:113172.

23. Costantini A, Mazzotti E. Italian validation of CoViD-19 Peritraumatic Distress Index and preliminary data in a sample of general population. Riv Psichiatr 2020;55:145-51.

24. Pedrozo-Pupo JC, Pedrozo-Cortés MJ, Campo-Arias A. Perceived stress associated with COVID-19 epidemic in Colombia: an online survey. Cad Saude Publica 2020;36:e00090520.

25. CHEN S-h, DAI J-m, HU Q, et al. Public anxiety and its influencing factors in the initial outbreak of COVID-19. JMS 2020;47:385-91.

26. Gómez-Salgado J, Andrés-Villas M, Domínguez-Salas S, Díaz-Milanés D, Ruiz-Frutos C. Related Health Factors of Psychological Distress During the COVID-19 Pandemic in Spain. Int J Environ Res Public Health. 2020;17:3947.

27. Forte G, Favieri F, Tambelli R, Casagrande M. The Enemy Which Sealed the World: Effects of COVID-19 Diffusion on the Psychological State of the Italian Population. J Clin Med 2020;9:E1802.

28. Wong LP, Alias H, Danaee M, et al. Uncovering psychobehavioral implications of SARS-CoV-2 infection in Iran. Transbound Emerg Dis 2020; published online Jun 8. Doi: 10.1111/tbed.13662.

29. Preis H, Mahaffey B, Heiselman C, Lobel M. Pandemic-related pregnancy stress and anxiety among women pregnant during the COVID-19 pandemic. Am J Obstet Gynecol MFM 2020; published online Jun 15. Doi: 10.1016/j.ajogmf.2020.100155.

30. Wu Y, Zhang C, Liu H, et al. Perinatal depressive and anxiety symptoms of pregnant women along with COVID-19 outbreak in China. Am J Obstet Gynecol 2020; published online May 11. Doi: 10.1016/j.ajog.2020.05.009.

31. Bruine de Bruin W. Age Differences in COVID-19 Risk Perceptions and Mental Health: Evidence From a National U.S. Survey Conducted in March 2020. J Gerontol B Psychol Sci Soc Sci 2020; published online May 29. Doi: 10.1093/geronb/gbaa074.

32. Kavčič T, Avsec A, Zager Kocjan G. Psychological Functioning of Slovene Adults during the COVID-19 Pandemic: Does Resilience Matter? Psychiatr Q 2020; published online Jun 17. Doi: 10.1007/s11126-020-09789-4.

33. Zhou S-J, Wang L-L, Yang R, et al. Sleep problems among Chinese adolescents and young adults during the coronavirus-2019 pandemic. Sleep Med 2020; published online Jun 6. Doi: 10.1016/j.sleep.2020.06.001.

34. Zhu Z, Liu Q, Jiang X, et al. The psychological status of people affected by the COVID-19 outbreak in China. J Psychiatr Res 2020;129:1-7.

35. Wang Y, Di Y, Ye J, Wei W. Study on the public psychological states and its related factors during the outbreak of coronavirus disease 2019 (COVID-19) in some regions of China. Psychol Health Med 2020; published online Mar 30. Doi: 10.1080/13548506.2020.1746817.

36. Qi R, Chen W, Liu S, et al. Psychological morbidities and fatigue in patients with confirmed COVID-19 during disease outbreak: prevalence and associated biopsychosocial risk factors. medRxiv 2020; published online May 11. Doi: 10.1101/2020.05.08.20031666.

37. Wang Y, Di Y, Ye J, Wei W. Study on the public psychological states and its related factors during the outbreak of coronavirus disease 2019 (COVID-19) in some regions of China. Psychol Health Med 2020; published online Mar 30. Doi: 10.1080/13548506.2020.1746817.

38. Zhou Q, Hu Z, Bian G, et al. Mental health and psychosocial function of general population during the COVID-19 epidemic in China. Clin Transl Med 2020; published online Jun 11. Doi: 10.1002/ctm2.103.

39. Tang F, Liang J, Zhang H, Kelifa MM, He Q, Wang P. COVID-19 related depression and anxiety among quarantined respondents. Psychol Health 2020; published online June 22. Doi: 10.1080/08870446.2020.1782410.

40. Verma S, Mishra A. Depression, anxiety, and stress and socio-demographic correlates among general Indian public during COVID-19. Int J Soc Psychiatry 2020; published online June 22. Doi: 10.1177/0020764020934508.

41. Gualano MR, Lo Moro G, Voglino G, Bert F, Siliquini R. Effects of Covid-19 Lockdown on Mental Health and Sleep Disturbances in Italy. Int J Environ Res Public Health 2020;17:E4779.

42. Kokou-Kpolou CK, Megalakaki O, Laimou D, Kousouri M. Insomnia during COVID-19 pandemic and lockdown: Prevalence, severity, and associated risk factors in French population. Psychiatry Res 2020;290:113128.

43. Mechili EA, Saliaj A, Kamberi F, et al. Is the mental health of young students and their family members affected during the quarantine period? Evidence from the COVID-19 pandemic in Albania. J Psychiatr Ment Health Nurs 2020; published online July 13. Doi: 10.1111/jpm.12672.

44. Lin Y, Hu Z, Alias H, Wong LP. Knowledge, Attitudes, Impact, and Anxiety Regarding COVID-19 Infection Among the Public in China. Front Public Health 2020;8:236.

45. Ueda M, Stickley A, Sueki H, Matsubayashi T. Mental Health Status of the General Population in Japan during the COVID-19 Pandemic. Psychiatry Clin Neurosci 2020; published online Jul 1. Doi: 10.1111/pcn.13105.

46. Naser AY, Dahmash EZ, Al-Rousan R, et al. Mental health status of the general population, healthcare professionals, and university students during 2019 coronavirus disease outbreak in Jordan: a cross-sectional study. Brain Behav 2020; published online Jun 24. Doi: 10.1002/brb3.1730.

47. Li LZ, Wang S. Prevalence and predictors of general psychiatric disorders and loneliness during COVID-19 in the United Kingdom. Psychiatry Res 2020;291:113267.

48. Fekih-Romdhane F, Ghrissi F, Abbassi B, Cherif W, Cheour M. Prevalence and predictors of PTSD during the COVID-19 pandemic: Findings from a Tunisian community sample. Psychiatry Res 2020;290:113131.

49. Shi L, Lu Z-A, Que J-Y, et al. Prevalence of and Risk Factors Associated With Mental Health Symptoms Among the General Population in China During the Coronavirus Disease 2019 Pandemic. JAMA Network Open 2020;3:e2014053-e.

50. Qi H, Liu R, Chen X, et al. Prevalence of Anxiety and Associated Factors for Chinese Adolescents during the COVID-19 Outbreak. Psychiatry Clin Neurosci 2020; published online Jul 2. Doi: 10.1111/pcn.13102.

51. Peng M, Mo B, Liu Y, et al. Prevalence, risk factors and clinical correlates of depression in quarantined population during the COVID-19 outbreak. J Affect Disord 2020;275:119-24.

52. Fu W, Wang C, Zou L, et al. Psychological health, sleep quality, and coping styles to stress facing the COVID-19 in Wuhan, China. Transl Psychiatry 2020;10:225.

53. Palgi Y, Shrira A, Ring L, et al. The loneliness pandemic: Loneliness and other concomitants of depression, anxiety and their comorbidity during the COVID-19 outbreak. J Affec Disord 2020;275:109-11.

54. S Seyahi E, Poyraz BC, Sut N, Akdogan S, Hamuryudan V. The psychological state and changes in the routine of the patients with rheumatic diseases during the coronavirus disease (COVID-19) outbreak in Turkey: a web-based cross-sectional survey. Rheumatol Int 2020;40:1229-38.

55. Lee Y, Yang BX, Liu Q, et al. The Synergistic Effect of Social Media Use and Psychological Distress on Depression in China During the COVID-19 Epidemic. Psychiatry Clin Neurosci 2020; published online Jul 1. Doi: 10.1111/pcn.13101.

56. Duan L, Shao X, Wang Y, et al. An investigation of mental health status of children and adolescents in china during the outbreak of COVID-19. J Affec Disord 2020;275:112-8.

57. Karatzias T, Shevlin M, Murphy J, et al. Posttraumatic Stress Symptoms and Associated Comorbidity During the COVID-19 Pandemic in Ireland: A Population-Based Study. J Trauma Stress 2020; published online Jul 13. Doi: 10.1002/jts.22565.

58. Liu X, Chen M, Wang Y, et al. Prenatal anxiety and obstetric decisions among pregnant women in Wuhan and Chongqing during the COVID-19 outbreak: a cross-sectional study. BJOG 2020; published online Jun 24. Doi: 10.1111/1471-0528.16381.

59. Yang Y, Zhu J-f, Yang S-y, et al. Prevalence and associated factors of poor sleep quality among Chinese returning workers during the COVID-19 pandemic. Sleep Med 2020; published online Jul 14. Doi: 10.1016/j.sleep.2020.06.034.

60. Hou T-Y, Mao X-F, Dong W, Cai W-P, Deng G-H. Prevalence of and factors associated with mental health problems and suicidality among senior high school students in rural China during the COVID-19 outbreak. Asian J Psychiatr 2020;54:102305.

61. Wang Z-H, Yang H-L, Yang Y-Q, et al. Prevalence of anxiety and depression symptom, and the demands for psychological knowledge and interventions in college students during COVID-19 epidemic: A large cross-sectional study. J Affect Disord 2020;275:188-93.

62. Ma Y-F, Li W, Deng H-B, et al. Prevalence of depression and its association with quality of life in clinically stable patients with COVID-19. J Affect Disord 2020;275:145-8.

63. Domínguez-Salas S, Gómez-Salgado J, Andrés-Villas M, Díaz-Milanés D, Romero-Martín M, Ruiz-Frutos C. Psycho-Emotional Approach to the Psychological Distress Related to the COVID-19 Pandemic in Spain: A Cross-Sectional Observational Study. Healthcare 2020;8:190.

64. Huang J, Liu F, Teng Z, et al. Public behavior change, perceptions, depression, and anxiety in relation to the COVID-19 outbreak. Open Forum Infect Dis 2020; published online Jul 3. Doi: 10.1093/ofid/ofaa273.

65. Liu D, Baumeister RF, Veilleux JC, et al. Risk Factors Associated with Mental Illness in Hospital Discharged Patients Infected with COVID-19 in Wuhan, China. Psychiatry Res 2020; published online Jul 13. Doi: 10.1016/j.psychres.2020.113297.

66. Ramasubramanian V, Mohandoss AA, Rajendhiran G, Pandian PRS, Ramasubramanian C. Statewide Survey of Psychological Distress Among People of Tamil Nadu in the COVID-19 Pandemic. Indian J Psychol Med 2020; published online Jul 6. Doi: 10.1177/0253717620935581.

67. Ben-Ezra M, Sun S, Hou WK, Goodwin R. The association of being in quarantine and related COVID-19 recommended and non-recommended behaviors with psychological distress in Chinese population. J Affect Disord 2020;275:66-8.

68. Mosli M, Alourfi M, Alamoudi A, Hashim A, Saadah O, Al Sulais E, et al. A cross-sectional survey on the psychological impact of the COVID-19 pandemic on inflammatory bowel disease patients in Saudi Arabia. Saudi J Gastroenterol. 2020; published online Jun 19. Doi: 10.4103/sjg.SJG_220_20.

69. World Health Organization [Internet]. Coronavirus disease 2019 (COVID-19) Situation Report – 40 [cited 2020 Aug 24]. Available from: https://www.who.int/docs/default-source/coronaviruse/situation-reports/20200229-sitrep-40-covid-19.pdf?sfvrsn=849d0665_2.

70. World Health Organization [Internet]. Countries - China. [cited 2020 Aug 24]. Available from: https://www.who.int/countries/chn/en/.

71. World Health Organization [Internet]. Countries - Vietnam. [cited 2020 Aug 24]. Available from: https://www.who.int/countries/vnm/en/.

72. Rothman K, Greenland S, Lash TL. Modern Epidemiology. 3rd ed. Lippincott Williams & Wilkins; 2008.
