# Supplementary material for: Factors associated with psychological distress during the coronavirus disease 2019 (COVID-19) pandemic on the predominantly general population: A systematic review and meta-analysis
Source: PLoS One. 2020 Dec 28;15(12):e0244630. doi: 10.1371/journal.pone.0244630 (PMC7769562; doi:10.1371/journal.pone.0244630)
Supplement: Attachment — Submitted filename: Response to Reviewers_.docx [file pone.0244630.s004.docx]

Manuscript ID: PONE-D-20-26693

Title: Factors associated with psychosocial disorders during the coronavirus disease 2019 (COVID-19) pandemic on the general population: a systematic review and meta-analysis

Dr. Michio Murakami,

Academic Editor, *PLOS ONE*

December 1, 2020

Dear Dr. Murakami,

We would like to thank the Editors and Reviewers for their insightful comments to our manuscript: “Factors associated with psychosocial disorders during the coronavirus disease 2019 (COVID-19) pandemic on the general population: a systematic review and meta-analysis” (PONE-D-20-26693). We have modified our manuscript accordingly and provided point-by-point response below. All changes are highlighted in yellow in this revision.

Thank you for the guidance to improve the quality of our manuscript. We sincerely hope that this revised manuscript meets the requirements of *PLOS ONE* and we look forward to hearing from you.

Sincerely yours,

(On behalf of the authors)

Tazeen H. Jafar, MD MPH

Duke-NUS Medical School, Singapore

Email: tazeen.jafar@duke-nus.edu.sg

***Journal Requirements:***

*When submitting your revision, we need you to address these additional requirements.*

*1. Please ensure that your manuscript meets PLOS ONE's style requirements, including those for file naming. The PLOS ONE style templates can be found at https://journals.plos.org/plosone/s/file?id=wjVg/PLOSOne_formatting_sample_main_body.pdf andhttps://journals.plos.org/plosone/s/file?id=ba62/PLOSOne_formatting_sample_title_authors_affiliations.pdf*

**Response:** Thank you for your comments. We have now ensured that our manuscript meets *PLOS ONE*’s style requirements, including those for filing name.

*2. Please clearly state your exclusion criteria.*

**Response:** We have now clearly stated the exclusion criteria in the manuscript: “Articles were excluded if they: 1) were not relevant (not using pre-defined factors as the exposure or psychological distress of COVID-19 as the outcome); 2) did not report the OR of factors (e.g. studies using linear regression analyses) or associated 95% CI; 3) were animal or experimental studies, reviews, or meta-analyses; 4) were conducted exclusively among healthcare professionals.” (Page 6, lines 104-108).

*3.Please include the funnel plot assessing publication bias in the Results section.*

**Response:** We have now included the funnel plot assessing publication bias as the journal required. If asymmetric funnel plot was observed on visual inspection which indicated publication bias and/or Egger’s t-test was significant (*P* <0.05), we further used the trim-and-fill method to examine the effect of publication bias on the associations. The results showed that studies on anxiety and depression with gender, age, and socioeconomic status (lower education, lower income) had enough power (included ten or more studies) to test for publication bias with funnel plots. Visual inspection of funnel plots revealed asymmetry for gender with anxiety and depression, and the Egger’s test was statistically significant (*P* ≤0.01). After applying the trim-and-fill method, the pooled RR was 1.08 (1.02-1.15) for anxiety and 1.06 (1.01-1.11) for depression, suggesting that the potential publication bias did not affect the significant associations of age with anxiety and depression. No significant asymmetry was observed for gender and SES strata with anxiety and depression, indicating the unlikelihood of publication bias for these factors. We have now updated the manuscript and presented the changes below:

**Methods:** “When data were available for ten or more studies, the publication bias was assessed by Egger’s regression (*P*<0.05 indicates statistical significance) and the funnel plot asymmetry [43]. If the potential publication bias exists, the trim-and-fill method was further used to assess the effect of publication bias [44].” (Page 8, lines 136-140)

**Results:** “**Assessment of publication bias.** Studies on anxiety and depression with gender, age, and SES strata (lower education, lower income) had enough power to test for publication bias with funnel plots (*n* ≥ten). Visual inspection of funnel plots revealed asymmetry for gender with anxiety and depression (S8 Fig), and the Egger’s test was statistically significant (*P* ≤0.01). After applying the trim-and-fill method, the pooled RR was 1.08 (1.02-1.15) for anxiety and 1.06 (1.01-1.11) for depression, suggesting that the potential publication bias did not affect the significant associations of age with anxiety and depression. No significant asymmetry was observed for gender and SES strata with anxiety and depression, indicating the unlikelihood of publication bias for these factors (S8 Fig).” (Page 22, lines 309-317)

**Discussion:** “Older age has been found to be associated with higher risk of COVID-19 infection [108]; however, it is interesting to observe that younger people (mainly <35 years) had higher odds of psychological distress during the COVID-19 pandemic. Although we observed an asymmetric funnel plot of age suggesting potential publication bias, the association of age with anxiety and depression remained statistically significant after applying the trim-and-fill method, indicating that the potential publication bias is unlikely to affect the observed associations of age with anxiety and depression.” (Page 24, lines 355-361)

**S8 Fig.** **Potential publication bias tested by funnel plots on gender, age, and socioeconomic status (education, income) with anxiety and depression.**

**Legend:** The dashed line is the pseudo 95% confidence intervals produced by the funnel plots. It corresponds to the 95% confidence interval for a given standard error. Egger’s test was significant for C) age and anxiety and D) age and depression (*P* ≤0.01). Egger’s test was not significant for other factors with anxiety and depression.

*4. Please summarize results for the heterogeneity tests and indicate whether heterogeneity was high or low across studies. Also, what were the implications of these results on the feasibility of undertaking a meta-analysis?*

**Response:** Thank you for your suggestions. We have now summarized results for the heterogeneity tests and whether heterogeneity was high or low across studies. We found that the heterogeneity was low in the association between current employment and anxiety (*I^2^*: 26.6%; *P*=0.22) and in the association of family/social support with both anxiety (*I^2^*: 0%; *P*=0.58) and depression (*I^2^*: 0%; *P*=0.58). In addition, we found that the heterogeneity was high in the remaining factors in association with anxiety and depression (*I^2^* range: 52.3%-95.1%, *P* <0.05).

For the implications of heterogeneity in undertaking a meta-analysis, a paper entitled “Reasons or excuses for avoiding meta-analysis in forest plots” published in *The BMJ* suggested that statistical heterogeneity alone is a weak indicator for avoiding quantitative synthesis; rather, appropriate quantitative methods may be considered to handle the heterogeneity (Ioannidis JPA, et al. BMJ. 2008; 336: 1413–1415). Therefore, to account for the between-study heterogeneity, we used DerSimonian and Laird random effects model instead of the fixed effects model to summarize the pooled odds ratio in the forest plots (DerSimonian R, et al. Control Clin Trials. 1986; 7: 177–88). We also conducted meta-regression (*P*<0.05 indicates statistical significance) and stratified analyses to explore the heterogeneity. According to the comments from the Editor and Reviewer 2, we stratified the results by study locations and psychometric instruments to explore potential influences of geographic differences and variations in instruments and cut-off points on the observed associations. As a result, we found that the point estimate of odds ratio observed in the overall population was largely consistent across all subgroups containing three or more studies, albeit the 95% CI became wider for subgroups with fewer studies, and did not achieve statistical significance for some groups with very few studies. However, except for the associations of anxiety with gender (higher odds in studies in Asian than in Europe, *P* for meta-regression=0.037), no statistically significant differences were found across subgroups of study locations (all *P*-values from meta-regression ≥0.09), indicating that geographic differences were less likely to influence the observed associations in the current meta-analysis. For psychometric instruments, only studies assessing the association between gender and anxiety offered enough power to have three subgroups, while other factors only had one subgroup; the meta-regression analysis suggested no statistical differences across subgroups of studies using different instruments or cut-off points (*P* for meta-regression=0.66). Nevertheless, we are aware that the small sample sizes and few numbers of subgroups for stratified analyses are limitations for the current study, and we have acknowledged them in the discussion. We have now added aforementioned changes in the manuscript, and also shown them below:

**Methods:** “We conducted meta-regression (*P* <0.05 indicates statistical significance) and stratified analyses by study locations and different instruments/cut-off points to evaluate the potential influence of geographic differences and variations in psychometric instrument and cut-off points on the results.” (Page 7, lines 130-133)

**Results:** “We further conducted stratified analyses to explore the heterogeneity. We found that the OR observed in the overall population was largely consistent across all subgroups by locations or instruments/cut-off points containing three or more studies, albeit the 95% CI became wider for subgroups with fewer studies and did not achieve statistical significance for subgroups with very few studies (S2 Table & S3 Table). Moreover, except for the association of anxiety with gender (higher odds in studies in Asian than in Europe, *P* for meta-regression=0.037), no statistically significant differences were found across subgroups of study locations for other factors (all *P*-values from meta-regression ≥0.09), indicating that geographic differences were less likely to influence the observed associations in the current meta-analysis (S2 Table). For psychometric instruments, only studies assessing the association between gender and anxiety offered enough power to have three subgroups (containing three or more studies), while other factors only had one subgroup. The meta-regression analysis suggested no statistical differences across subgroups of studies using different instruments or cut-off points (*P*-value from meta-regression =0.66) (S3 Table).” (Page 21, lines 291-304)

**Discussions:** “Furthermore, the data points vary for the different outcomes, and we observed a substantial heterogeneity for most of the associations. However, we have included all published studies reporting the association for the outcomes of interest during the pandemic period covered during our study. In addition, we conducted meta-regression and stratified analyses by study locations and different instruments and cut-off points, and observed largely non-significant *P*-values for meta-regression analyses and consistent point estimates of OR across subgroups, suggesting no substantial statistical differences across subgroups. Nevertheless, the sample sizes and numbers of subgroups were small for the stratified analyses, thus the 95% CI became wider and did not achieve statistical significance for subgroups with very few studies; future studies with larger sample sizes are warranted to validate our findings.” (Page 28, lines 454-464)

**S2 Table. Stratified analyses of factors and risks of anxiety and depression by study locations.**

|  | **Anxiety** | | | |  | **Depression** | | | |  |
| --- | --- | --- | --- | --- | --- | --- | --- | --- | --- | --- |
|  | **OR (95% CI)^a^** | ***N* of studies** | ***I*^2^ (%)** | **P for heterogeneity** | **P for meta-regression** | **RR (95% CI)^a^** | ***N* of studies** | ***I*^2^ (%)** | **P for heterogeneity** | **P for meta-regression** |
| **Gender (women vs. men)** | |  |  |  |  |  |  |  |  |  |
| Overall results | 1.48 (1.29-1.71) | 29 | 90.8% | <0.001 |  | 1.16 (1.07-1.26) | 25 | 75.0% | <0.001 |  |
| Stratified by locations |  |  |  |  | 0.037 |  |  |  |  | 0.98 |
| Asia | 1.26 (1.01-1.57) | 11 | 90.6% | <0.001 |  | 1.11 (0.96-1.29) | 15 | 84.6% | <0.001 |  |
| Middle East | - | 2 | - | - |  | - | 2 | - | - |  |
| Europe | 1.12 (0.93-1.34) | 4 | 15.4% | 0.14 |  | - | 2 | - | - |  |
| U.S. | - | 1 | - | - |  | - | 0 | - | - |  |
| **Age (younger vs. older)** | |  |  |  |  |  |  |  |  |  |
| Overall results | 1.20 (1.13-1.26) | 21 | 91.7% | <0.001 |  | 1.13 (1.08-1.18) | 18 | 95.1% | <0.001 |  |
| Stratified by locations |  |  |  |  | 0.87 |  |  |  |  | 0.40 |
| Asia | 1.36 (1.19-1.56) | 13 | 91.3% | <0.001 |  | 1.25 (1.12-1.40) | 13 | 95.9% | <0.001 |  |
| Middle East | 1.23 (0.86-1.76) | 3 | 76.7% | <0.001 |  | - | 2 | - | - |  |
| Europe | 1.10 (1.03-1.18) | 4 | 93.3% | <0.001 |  | 1.02 (0.99-1.06) | 3 | 89.8% | <0.001 |  |
| U.S. | - | 0 | - | - |  | - | 0 | - | - |  |
| **Education (lower vs. higher)** | |  |  |  |  |  |  |  |  |  |
| Overall results | 1.21 (1.05-1.40) | 18 | 86.1% | <0.001 |  | 1.15 (1.03-1.29) | 20 | 82.0% | 0.016 |  |
| Stratified by locations |  |  |  |  | 0.81 |  |  |  |  | 0.27 |
| Asia | 1.26 (1.01-1.57) | 11 | 90.6% | <0.001 |  | 1.11 (0.96-1.29) | 15 | 84.6% | <0.001 |  |
| Middle East | - | 2 | - | - |  | - | 2 | - | - |  |
| Europe | 1.12 (0.93-1.34) | 4 | 45.4% | 0.14 |  | - | 2 | - | - |  |
| U.S. | - | 1 | - | - |  | - | 1 | - | - |  |
| **Income (lower vs. higher)** | |  |  |  |  |  |  |  |  |  |
| Overall results | 1.45 (1.24-1.69) | 13 | 82.3% | <0.001 |  | 1.56 (1.26-1.92) | 10 | 85.4% | <0.001 |  |
| Stratified by locations |  |  |  |  | 0.69 |  |  |  |  | 0.62 |
| Asia | 1.46 (1.20-1.78) | 9 | 87.3% | <0.001 |  | 1.47 (1.12-1.91) | 7 | 89.0% | <0.001 |  |
| Middle East | - | 1 | - | - |  | - | 1 | - | - |  |
| Europe | - | 1 | - | - |  | - | 1 | - | - |  |
| U.S. | - | 2 | - | - |  | - | 1 | - | - |  |
| **Current employment (yes vs. no)** | |  |  |  |  |  |  |  |  |  |
| Overall results | 0.89 (0.78-1.02) | 8 | 26.6% | 0.22 |  | 0.76 (0.61-0.95) | 7 | 63.8% | 0.011 |  |
| Stratified by locations |  |  |  |  | 0.31 |  |  |  |  | 0.42 |
| Asia | 0.87 (0.76-1.00) | 4 | 0% | 0.48 |  | 0.95 (0.61-1.49) | 3 | 80% | 0.007 |  |
| Middle East | - | 2 | - | - |  | - | 2 | - | - |  |
| Europe | - | 1 | - | - |  | - | 1 | - | - |  |
| U.S. | - | 1 | - | - |  | - | 1 | - | - |  |
| **Residential area (rural vs. urban)** | |  |  |  |  |  |  |  |  |  |
| Overall results | 1.13 (1.00-1.29) | 7 | 82.9% | <0.001 |  | 0.98 (0.85-1.12) | 7 | 81.6% | <0.001 |  |
| Stratified by locations |  |  |  |  | 0.11 |  |  |  |  | 0.15 |
| Asia | 1.11 (0.98-1.25) | 6 | 83.2% | <0.001 |  | 0.95 (0.83-1.08) | 6 | 81.9% | <0.001 |  |
| Middle East | - | 0 | - | - |  | - | 0 | - | - |  |
| Europe | - | 1 | - | - |  | - | 1 | - | - |  |
| U.S. | - | 0 | - | - |  | - | 0 | - | - |  |
| **Suspected/confirmed COVID-19 cases (yes vs. no)** | | |  |  |  |  |  |  |  |  |
| Overall results | 1.70 (1.41-2.06) | 15 | 79.5% | <0.001 |  | 1.84 (1.39-2.43) | 8 | 73.5% | <0.001 |  |
| Stratified by locations |  |  |  |  | 0.66 |  |  |  |  | 0.58 |
| Asia | 1.91 (1.50-2.42) | 10 | 60.7% | 0.006 |  | 1.98 (1.47-2.67) | 6 | 57.9% | 0.036 |  |
| Middle East | - | 2 | - | - |  | - | 1 | - | - |  |
| Europe | - | 2 | - | - |  | - | 1 | - | - |  |
| U.S. | - | 1 | - | - |  | - | 0 | - | - |  |
| **Living in the hard-hit area (yes vs. no)** | |  |  |  |  |  |  |  |  |  |
| Overall results | 1.57 (1.36-1.81) | 10 | 73.9% | <0.001 |  | 1.33 (1.16-1.53) | 10 | 69.1% | 0.001 |  |
| Stratified by locations |  |  |  |  | 0.47 |  |  |  |  | 0.68 |
| Asia | 1.61 (1.38-1.88) | 9 | 73.0% | <0.001 |  | 1.31 (1.11-1.54) | 9 | 72.4% | <0.001 |  |
| Middle East | - | 0 | - | - |  | - | 0 | - | - |  |
| Europe | - | 0 | - | - |  | - | 0 | - | - |  |
| U.S. | - | 1 | - | - |  | - | 1 | - | - |  |
| **Pre-existing physical conditions (yes vs. no)** | |  |  |  |  |  |  |  |  |  |
| Overall results | 1.48 (1.21-1.81) | 11 | 65.2% | 0.001 |  | 1.42 (1.12-1.80) | 11 | 89.0% | <0.001 |  |
| Stratified by locations |  |  |  |  | 0.09 |  |  |  |  | 0.11 |
| Asia | - | 2 | - | - |  | 1.66 (1.12-2.45) | 5 | 93.7% | <0.001 |  |
| Middle East | 0.98 (0.63-1.53) | 3 | 29.9% | 0.24 |  | - | 2 | - | - |  |
| Europe | 1.60 (1.19-2.14) | 5 | 68.3% | 0.013 |  | 1.44 (1.04-1.98) | 4 | 75.4% | 0.007 |  |
| U.S. | - | 1 | - | - |  | - | 0 | - | - |  |
| **Pre-existing mental conditions (yes vs. no)** | |  |  |  |  |  |  |  |  |  |
| Overall results | 1.82 (1.34-2.48) | 6 | 70.8% | 0.004 |  | 1.75 (0.98-3.14) | 6 | 93.5% | <0.001 |  |
| Stratified by locations |  |  |  |  | 0.13 |  |  |  |  | 0.38 |
| Asia | - | 2 | - | - |  | - | 2 | - | - |  |
| Middle East | 1.57 (0.90-2.73) | 3 | 68.0% | 0.044 |  | 1.34 (0.75-2.41) | 3 | 83.1% | 0.003 |  |
| Europe | - | 0 | - | - |  | - | 0 | - | - |  |
| U.S. | - | 1 | - | - |  | - | 1 | - | - |  |
| **Media exposure (longer vs. shorter)** | |  |  |  |  |  |  |  |  |  |
| Overall results | 1.57 (1.16-2.13) | 6 | 94.5% | <0.001 |  | 1.34 (1.12-1.60) | 8 | 86.2% | <0.001 |  |
| Stratified by locations |  |  |  |  | 0.17 |  |  |  |  | 0.47 |
| Asia | 1.80 (1.36-2.38) | 4 | 82.9% | 0.001 |  | 1.43 (1.13-1.80) | 6 | 79.4% | <0.001 |  |
| Middle East | - | 0 | - | - |  | - | 0 | - | - |  |
| Europe | - | 2 | - | - |  | - | 2 | - | - |  |
| U.S. | - | 0 | - | - |  | - | 0 | - | - |  |
| **Social/family support (yes vs. no)** | |  |  |  |  |  |  |  |  |  |
| Overall results | 0.68 (0.58-0.79) | 4 | 0% | 0.58 |  | 0.47 (0.40-0.56) | 5 | 0% | 0.50 |  |
| Stratified by locations |  |  |  |  | 0.89 |  |  |  |  | 0.42 |
| Asia | 0.69 (0.57-0.83) | 3 | 0% | 0.54 |  | 0.43 (0.34-0.55) | 4 | 0% | 0.54 |  |
| Middle East | - | 0 | - | - |  | - | 0 | - | - |  |
| Europe | - | 0 | - | - |  | - | 0 | - | - |  |
| U.S. | - | 1 | - | - |  | - | 1 | - | - |  |
| **Physical activity (longer vs. shorter)** | |  |  |  |  |  |  |  |  |  |
| Overall results | 0.71 (0.58-0.88) | 5 | 52.3% | 0.08 |  | 0.69 (0.50-0.94) | 6 | 84.8% | <0.001 |  |
| Stratified by locations |  |  |  |  | - |  |  |  |  | - |
| Asia | 0.71 (0.58-0.88) | 5 | 52.3% | 0.08 |  | 0.69 (0.50-0.94) | 6 | 84.8% | <0.001 |  |
| Middle East | - | 0 | - | - |  | - | 0 | - | - |  |
| Europe | - | 0 | - | - |  | - | 0 | - | - |  |
| U.S. | - | 0 | - | - |  | - | 0 | - | - |  |

**Abbreviations:** OR, odds ratio; CI, confidence interval.

^a^The odds ratios were calculated using the random-effects model among studies with three or more data points.

**S3 Table. Sensitivity analyses of factors and risks of anxiety and depression among studies using the same instrument and cut-off values.**

|  | **Anxiety** | | | |  |  | **Depression** | | | |  |
| --- | --- | --- | --- | --- | --- | --- | --- | --- | --- | --- | --- |
|  | **OR**  **(95% CI)^a^** | ***N* of studies** | ***I*^2^ (%)** | **P for heterogeneity** | **P for meta-regression** |  | **RR**  **(95% CI)^a^** | ***N* of studies** | ***I*^2^ (%)** | **P for heterogeneity** | **P for meta-regression** |
| **Gender (women vs. men)** | |  |  |  | 0.66 | **Age (younger vs. older)** | |  |  |  | - |
| Overall results | 1.20  (1.13-1.26) | 21 | 91.7% | <0.001 |  | Overall results | 1.13  (1.08-1.18) | 18 | 95.1% | <0.001 |  |
| Use GAD-7 ≥10 | 1.13  (0.93-1.38) | 6 | 68.6% | 0.007 |  | Use PHQ-9 ≥10 | 1.06  (0.98-1.16) | 5 | 92.1% | <0.001 |  |
| Use GAD-7 ≥5 | 1.33  (0.91-1.93) | 5 | 69.6% | <0.001 |  |  |  |  |  |  |  |
| Use GAD-2 ≥3 | 1.55  (1.07-2.24) | 3 | 76.2% | 0.015 |  |  |  |  |  |  |  |
| **Age (younger vs. older)** | |  |  |  | **-** | **Gender (women vs. men)** | |  |  |  | - |
| Overall results | 1.48  (1.29-1.71) | 29 | 90.8% | <0.001 |  | Overall results | 1.16  (1.07-1.26) | 25 | 75.0% | <0.001 |  |
| Use GAD-7 ≥10 | 1.17  (1.03-1.34) | 10 | 76.4% | <0.001 |  | Use PHQ-9 ≥10 | 1.13  (0.93-1.38) | 6 | 68.6% | 0.007 |  |
| **Education (lower vs. higher)** | |  |  |  | **-** | **Education (lower vs. higher)** | |  |  |  | - |
| Overall results | 1.21  (1.05-1.40) | 18 | 86.1% | <0.001 |  | Overall results | 1.15  (1.03-1.29) | 20 | 82.0% | 0.016 |  |
| Use GAD-7 ≥10 | 1.51  (0.98-2.35) | 5 | 91.4% | <0.001 |  | Use PHQ-9 ≥10 | 1.08  (0.80-1.47) | 5 | 82.5% | <0.001 |  |
| **Income (lower vs. higher)** | |  |  |  | - |  |  |  |  |  |  |
| Overall results | 1.45  (1.24-1.69) | 13 | 82.3% | <0.001 |  |  |  |  |  |  |  |
| Use GAD-7 ≥5 | 1.65  (0.75-3.62) | 3 | 96.7% | <0.001 |  |  |  |  |  |  |  |
| **Living in the hard-hit area (yes vs. no)** | | |  |  | - |  |  |  |  |  |  |
| Overall results | 1.57  (1.36-1.81) | 10 | 73.9% | <0.001 |  |  |  |  |  |  |  |
| Use GAD-7 ≥10 | 1.68  (1.16-2.43) | 4 | 77.9% | 0.004 |  |  |  |  |  |  |  |
| **Suspected/confirmed COVID-19 cases (yes vs. no)** | | | |  | - |  |  |  |  |  |  |
| Overall results | 1.70  (1.41-2.06) | 15 | 79.5% | <0.001 |  |  |  |  |  |  |  |
| Use GAD-2 ≥3 | 1.75  (1.15-2.66) | 3 | 67.3% | 0.047 |  |  |  |  |  |  |  |
| **Pre-existing physical conditions (yes vs. no)** | | | |  | - |  |  |  |  |  |  |
| Overall results | 1.48  (1.21-1.81) | 11 | 65.2% | 0.001 |  |  |  |  |  |  |  |
| Use GAD-7 ≥10 | 1.26  (0.79-2.00) | 3 | 73.9% | 0.022 |  |  |  |  |  |  |  |
| **Physical activity (longer vs. shorter)** | | |  |  | - |  |  |  |  |  |  |
| Overall results | 0.71  (0.58-0.88) | 5 | 52.3% | 0.08 |  |  |  |  |  |  |  |
| Use GAD-7 ≥5 | 0.80  (0.65-0.98) | 3 | 34.7% | 0.22 |  |  |  |  |  |  |  |

**Abbreviations:** OR, odds ratio; CI, confidence interval.

^a^The odds ratios were calculated using the random-effects model among studies with three or more data points.

*5.We note that you have indicated that data from this study are available upon request. PLOS only allows data to be available upon request if there are legal or ethical restrictions on sharing data publicly. For information on unacceptable data access restrictions, please see http://journals.plos.org/plosone/s/data-availability#loc-unacceptable-data-access-restrictions.*

*In your revised cover letter, please address the following prompts:*

*a) If there are ethical or legal restrictions on sharing a de-identified data set, please explain them in detail (e.g., data contain potentially identifying or sensitive patient information) and who has imposed them (e.g., an ethics committee). Please also provide contact information for a data access committee, ethics committee, or other institutional body to which data requests may be sent.*

*b) If there are no restrictions, please upload the minimal anonymized data set necessary to replicate your study findings as either Supporting Information files or to a stable, public repository and provide us with the relevant URLs, DOIs, or accession numbers. Please see http://www.bmj.com/content/340/bmj.c181.long for guidelines on how to de-identify and prepare clinical data for publication. For a list of acceptable repositories, please see http://journals.plos.org/plosone/s/data-availability#loc-recommended-repositories.*

*We will update your Data Availability statement on your behalf to reflect the information you provide.*

**Response:** Thank you for your comment. We have now uploaded the anonymized dataset as a Supporting Information file entitled “Anonymous dataset for analyses to produce forest plots”.

*6. We note that [Figure(s) 2] in your submission contain [map/satellite] images which may be copyrighted. All PLOS content is published under the Creative Commons Attribution License (CC BY 4.0), which means that the manuscript, images, and Supporting Information files will be freely available online, and any third party is permitted to access, download, copy, distribute, and use these materials in any way, even commercially, with proper attribution. For these reasons, we cannot publish previously copyrighted maps or satellite images created using proprietary data, such as Google software (Google Maps, Street View, and Earth). For more information, see our copyright guidelines: http://journals.plos.org/plosone/s/licenses-and-copyright. We require you to either (1) present written permission from the copyright holder to publish these figures specifically under the CC BY 4.0 license, or (2) remove the figures from your submission:*

**Response:** Thank you for your comment. We have now removed the potential copyrighted figure sourced from the World Health Organization from our Figure 2. The rest of Figure 2 was made by authors. The updated figure is shown below for your ease of reference.

***Additional Editor Comments (if provided):***

*1. As pointed out by reviewer 1, if there are previous studies related to systematic reviews and meta-analysis, the statement in L362-364 needs to be revised.*

**Response:** Thank you very much for your comments. As the reviewer 1 mentioned, there are a few additional systematic reviews and meta-analyses examining the psychological distress during COVID-19, which were published after the submission of the current manuscript to PLOS ONE. We have now included these meta-analyses in our manuscript. These studies mainly focused on examining the prevalence of psychological distress, while the primary objective of the current study was to identify factors associated with higher psychological distress during COVID-19 using quantitative analyses. We have now revised the statement and highlighted our novelty accordingly:

**Discussion:** “Previous meta-analyses among general population mainly examined the prevalence of psychological distress [6, 32-37]. In comparison, the current meta-analysis also performed quantitative assessment and reported factors associated with psychological distress during the COVID-19 pandemic in the predominantly general population. Nevertheless, we are not aware of other meta-analyses assessing factors of psychological distress in the general population.” (Pages 26-27, lines 422-427)

*2. Please add the cut-off points applied to the indicators in each study in Table 1.*

**Response:** We have now added the cut-off points of the indicators for each study in Table 1 (Pages 10-16). Due to the length of the table, we did not paste it here in the response letter. Please kindly refer to the manuscript for the changes.

*3. The various indicators and cut-off points were analyzed together in this study. The strength of associations between mental health outcomes and factors can depend on the types of indicators and cut-off points. In discussing the strength of the association with each factor, the authors need to add further analyses to examine whether there are differences across indicators and cut-off points. If necessary, please perform a stratified analysis for each of the same indicators with the same cut-off points to deepen the discussion.*

**Response:** Thank you for your comments. We agree with the Editor that different types of indicators and cut-off points may bring potential heterogeneity to the study results. As we mentioned in the response to journal requirement, we have now further conducted stratified analyses and meta-regression to examine the potential impact of variations in indicators and cut-off points on the study results. We found that among all factors, studies assessing the association between gender and anxiety had enough statistical power to have three subgroups (containing three or more studies), while other factors only had one subgroup. The point estimate of odds ratio observed in the overall population was largely consistent across all subgroups, albeit the 95% CI became wider for subgroups with fewer studies and did not achieve statistical significance for some groups with very few studies. In addition, the meta-regression analysis suggested no statistical differences across subgroups of studies using different instruments or cut-off points (*P*-value from meta-regression =0.66). Nevertheless, we are aware that the small sample sizes and few numbers of subgroups for stratified analyses are limitations for the current study, and we have acknowledged them in the discussion. We have now added the changes in the manuscript, and shown them below:

**Methods:** “We conducted meta-regression (*P* <0.05 indicates statistical significance) and stratified analyses by … different instruments/cut-off points to evaluate the potential influence of … variations in psychometric instrument and cut-off points on the results.” (Page 7, lines 130-133)

**Results:** “We further conducted stratified analyses to explore the heterogeneity. We found that the point estimate of OR observed in the overall population was largely consistent across all subgroups by locations or instruments/cut-off points with three or more studies, albeit the 95% CI became wider for subgroups with fewer studies, and did not achieve statistical significance for some groups with very few studies (S2 Table & S3 Table). … For psychometric instruments, only studies assessing the association between gender and anxiety offered enough power to have three subgroups, while other factors only had one subgroup. The meta-regression analysis suggested no statistical differences across subgroups of studies using different instruments or cut-off points (*P*-value from meta-regression =0.66) (S3 Table).” (Page 21, lines 291-304)

**Discussions:** “Fourth, instruments to measure indicators of psychological distress (e.g. anxiety, depression) were not identical in all studies. However, we only included studies that used standardized and validated instruments to measure psychological distress, which mitigates the possibility of systematic bias to our best extent. Furthermore, the data points vary for the different outcomes, and we observed a substantial heterogeneity for most of the associations. However, we have included all published studies reporting the association for the outcomes of interest during the pandemic period covered during our study. In addition, we conducted meta-regression and stratified analyses by study locations and different instruments and cut-off points, and observed largely non-significant *P*-values for meta-regression analyses and consistent point estimates of OR across subgroups, suggesting no substantial statistical differences across subgroups. Nevertheless, the sample sizes and numbers of subgroups were small for the stratified analyses, thus the 95% CI became wider and did not achieve statistical significance for subgroups with very few studies; future studies with larger sample sizes are warranted to validate our findings.” (Page 28, lines 451-464)

**S3 Table. Sensitivity analyses of factors and risks of anxiety and depression among studies using the same instrument and cut-off values.**

|  | **Anxiety** | | | |  |  | **Depression** | | | |  |
| --- | --- | --- | --- | --- | --- | --- | --- | --- | --- | --- | --- |
|  | **OR**  **(95% CI)^a^** | ***N* of studies** | ***I*^2^ (%)** | **P for heterogeneity** | **P for meta-regression** |  | **RR**  **(95% CI)^a^** | ***N* of studies** | ***I*^2^ (%)** | **P for heterogeneity** | **P for meta-regression** |
| **Gender (women vs. men)** | |  |  |  | 0.66 | **Age (younger vs. older)** | |  |  |  | - |
| Overall results | 1.20  (1.13-1.26) | 21 | 91.7% | <0.001 |  | Overall results | 1.13  (1.08-1.18) | 18 | 95.1% | <0.001 |  |
| Use GAD-7 ≥10 | 1.13  (0.93-1.38) | 6 | 68.6% | 0.007 |  | Use PHQ-9 ≥10 | 1.06  (0.98-1.16) | 5 | 92.1% | <0.001 |  |
| Use GAD-7 ≥5 | 1.33  (0.91-1.93) | 5 | 69.6% | <0.001 |  |  |  |  |  |  |  |
| Use GAD-2 ≥3 | 1.55  (1.07-2.24) | 3 | 76.2% | 0.015 |  |  |  |  |  |  |  |
| **Age (younger vs. older)** | |  |  |  | **-** | **Gender (women vs. men)** | |  |  |  | - |
| Overall results | 1.48  (1.29-1.71) | 29 | 90.8% | <0.001 |  | Overall results | 1.16  (1.07-1.26) | 25 | 75.0% | <0.001 |  |
| Use GAD-7 ≥10 | 1.17  (1.03-1.34) | 10 | 76.4% | <0.001 |  | Use PHQ-9 ≥10 | 1.13  (0.93-1.38) | 6 | 68.6% | 0.007 |  |
| **Education (lower vs. higher)** | |  |  |  | **-** | **Education (lower vs. higher)** | |  |  |  | - |
| Overall results | 1.21  (1.05-1.40) | 18 | 86.1% | <0.001 |  | Overall results | 1.15  (1.03-1.29) | 20 | 82.0% | 0.016 |  |
| Use GAD-7 ≥10 | 1.51  (0.98-2.35) | 5 | 91.4% | <0.001 |  | Use PHQ-9 ≥10 | 1.08  (0.80-1.47) | 5 | 82.5% | <0.001 |  |
| **Income (lower vs. higher)** | |  |  |  | - |  |  |  |  |  |  |
| Overall results | 1.45  (1.24-1.69) | 13 | 82.3% | <0.001 |  |  |  |  |  |  |  |
| Use GAD-7 ≥5 | 1.65  (0.75-3.62) | 3 | 96.7% | <0.001 |  |  |  |  |  |  |  |
| **Living in the hard-hit area (yes vs. no)** | | |  |  | - |  |  |  |  |  |  |
| Overall results | 1.57  (1.36-1.81) | 10 | 73.9% | <0.001 |  |  |  |  |  |  |  |
| Use GAD-7 ≥10 | 1.68  (1.16-2.43) | 4 | 77.9% | 0.004 |  |  |  |  |  |  |  |
| **Suspected/confirmed COVID-19 cases (yes vs. no)** | | | |  | - |  |  |  |  |  |  |
| Overall results | 1.70  (1.41-2.06) | 15 | 79.5% | <0.001 |  |  |  |  |  |  |  |
| Use GAD-2 ≥3 | 1.75  (1.15-2.66) | 3 | 67.3% | 0.047 |  |  |  |  |  |  |  |
| **Pre-existing physical conditions (yes vs. no)** | | | |  | - |  |  |  |  |  |  |
| Overall results | 1.48  (1.21-1.81) | 11 | 65.2% | 0.001 |  |  |  |  |  |  |  |
| Use GAD-7 ≥10 | 1.26  (0.79-2.00) | 3 | 73.9% | 0.022 |  |  |  |  |  |  |  |
| **Physical activity (longer vs. shorter)** | | |  |  | - |  |  |  |  |  |  |
| Overall results | 0.71  (0.58-0.88) | 5 | 52.3% | 0.08 |  |  |  |  |  |  |  |
| Use GAD-7 ≥5 | 0.80  (0.65-0.98) | 3 | 34.7% | 0.22 |  |  |  |  |  |  |  |

**Abbreviations:** OR, odds ratio; CI, confidence interval.

^a^The odds ratios were calculated using the random-effects model among studies with three or more data points.

***Reviewer #1:***

*This paper is a meta-analytic review of the factors associated with "psychosocial disorders" in the context of the COVID-19 pandemic. There are several major problems with this paper in its current form:*

*1. The term "psychosocial disorders" is conceptually flawed. A "psychological disorder" (synonym: "mental disorder") is a medical-psychological term referring to a specific set of symptoms associated with mental distress and dysfunction. "Social disorder" is a general term referring to disturbances in social order such as civil unrest, famine or violence. To conflate the* *two and invent a new term ("psychosocial disorders") is erroneous.*

**Response:** Thank you very much for your comments. We have now changed the term “psychosocial disorders” to “psychological distress” throughout the manuscript as suggested in your third comment.

*2. The paper itself has only examined psychological disorders (as mentioned in the Abstract, the primary outcome measures are "anxiety" and "depression") and not social disorders such as poverty, job loss, civil unrest or lack of access to healthcare. Hence, the paper may be best given a new title using the term "psychological disorder" or "mental disorder". Yet even these terms are not without their problems in the context of this paper, as discussed immediately below.*

**Response:** We agree with the reviewer that our paper examined the psychological disorders rather than the social disorders. As we mentioned in the response to your first comment, we have changed the term “psychosocial disorders” to “psychological distress” throughout the manuscript as suggested in your third comment.

*3. The diagnosis of a psychological disorder is a formal process requiring the application of operationalized diagnostic criteria (such as the DSM-5) by a trained mental health care or allied professional. Though there are brief instruments, such as checklists or self-rated questionnaires, that can aid in this process, these are only screening instruments, and anyone screening positive on such an instrument would still need to have their diagnosis confirmed using the standard criteria mentioned above. The vast majority (if not all) of the studies included in this paper have only made use of screening instruments. Hence, strictly speaking, these studies cannot be used to estimate the frequency of "psychological disorder"; they can only assess the frequency of "symptoms of psychological disorder" (such as "depressive symptoms" or "anxiety symptoms"), or - to use a broad but still acceptable term - "psychological distress". Hence, "psychological distress" or "symptoms of X/Y/Z" would be an ideal term to use in the title and text of this article.*

**Response:** Thank you for the detailed explanation. We agree with the reviewer that majority of studies used these instruments as screening tools for the symptoms of psychological disorder. Therefore, we changed “psychosocial disorders” to “psychological distress” throughout our manuscript and highlighted in yellow.

*4. To my knowledge, there are already no less than three meta-analytic papers addressing the exact question that this paper examines - Deng et al. 2020, Bueno-Notivol et al. 2020, and Krishnamoorthy et al. 2020. This is not counting three meta-analyses exclusive to healthcare workers and other related papers. Hence, the need for another paper of this kind is unclear, especially since the authors of this paper have not acknowledged even one of these earlier meta-analyses in their own work. Unless the current paper represents a significant improvement or advance over these papers, either chronologically or in terms of methodology or quality, I see little justification for an unnecessary replication.*

**Response:** Thank you for your valuable insights. We agree with the reviewer that it is important to highlight the novelty of our paper. The three studies mentioned by the reviewer were published after the submission of the current manuscript to PLOS ONE. We have now mentioned them in our revised manuscript. We also identified two additional relevant meta-analyses and have cited them as well. All these existing meta-analyses only examined the prevalence of psychological distress among predominantly general population (contain a small subset of healthcare professionals and patients). The novelty of our study was to examine factors of psychological distress using quantitative analyses and forest plots. We have now further clarified the justification in the manuscript and shown them below:

**Introduction:** “Although a few meta-analyses have been conducted to investigate the prevalence of psychological distress related to the COVID-19 pandemic, studies on determinants of psychological distress have largely focused on healthcare workers [6, 23-26]. Systematic reviews on factors associated with psychological distress in the general population during the COVID-19 pandemic have not been reported.” (Page 4, lines 62-67)

**Discussion:** “Previous meta-analyses among general population only reported the prevalence of psychological distress [6, 32-37]. In comparison, the primary objective of the current meta-analysis was to determine the factors associated with psychological distress using quantitative assessment during the COVID-19 pandemic in the predominantly general population. We are not aware of other meta-analyses assessing factors of psychological distress in the general population.” (Pages 26-27, lines 422-427)

*5. The authors have used the term "general population" in their title and text to define their target population: however, they have gone on to include studies of high-risk or vulnerable populations such as the mentally ill, those with epilepsy, pregnant women, and healthcare workers! There is little justification for introducing so much additional heterogeneity into what is already a very diverse and heterogeneous group of general population studies, especially since these groups already have higher prior rates of psychological distress. The study would be significantly improved in quality by a focus on general population studies alone.*

**Response:** Thank you for your comments. Since the high-risk and vulnerable groups (pregnant women, those with pre-existing condition such as epilepsy) are also part of the general population, we included them in our study. In addition, studies on predominantly general population with healthcare providers were also included. However, studies on exclusively healthcare providers were excluded. We have clarified the title of our manuscript to “predominantly general population” to be more reflective of our study population.

In addition, we further conducted sensitivity analyses and repeated all analyses excluding 11 studies containing high-risk or vulnerable populations, or studies using random sampling techniques and thus containing a small subset of healthcare workers. We found that the significant associations remained essentially the same for all factors, indicating that the additional inclusion of these populations did not impact our results. The results of the sensitivity analysis are now reported in the revised manuscript at Page 21, lines 306-309 and S4 Table in the Appendix.

**Introduction:** “Although a few meta-analyses have been conducted to investigate the prevalence of psychological distress related to the COVID-19 pandemic, studies on determinants of psychological distress have largely focused on healthcare workers [4, 11-14]. Systematic reviews on factors associated with psychological distress in the general population during the COVID-19 pandemic have not been reported.” (Page 4, lines 62-67)

“Therefore, we conducted a systematic review and meta-analysis to explore factors associated with psychological distress among the predominantly general population including high-risk or vulnerable patients with particular focus on gender, age, rural residence, and SES strata. We hypothesize that women, older adults, individuals residing in rural versus urban areas, and those of lower SES strata are associated with higher odds of psychological distress during the COVID-19 pandemic.” (Page 5, lines 73-78)

**Methods:** “Articles were excluded if they: 1) were not relevant (not using pre-defined factors as the exposure or psychological distress of COVID-19 as the outcome); 2) did not report the OR of factors (e.g. studies using linear regression analyses) or associated 95% CI; 3) were animal or experimental studies, reviews, or meta-analyses; 4) were conducted exclusively among healthcare professionals.” (Page 6, lines 104-108)

“In the sensitivity analyses, we further repeated the analyses excluding studies containing high-risk or vulnerable populations, or studies using random sampling techniques and thus containing a small subset of healthcare workers.” (Page 7, lines 133-136)

**Results:** “**Sensitivity analyses.** We further repeated the aforementioned analyses excluding 11 studies containing high-risk or vulnerable populations, or a small subset of healthcare workers. We found that all significant associations remained the same across all factors, indicating that the heterogeneity in populations did not impact our results (S4 Table).” (Page 21, lines 305-308)

**Discussion:** “Second, we included a predominantly general population for the current meta-analysis, as well as a few studies among high-risk and vulnerable patients, and a small subset of healthcare workers, which may bring potential heterogeneity to our results. Nevertheless, we conducted sensitivity analyses among studies only containing general populations, and found the same significant results across all factors, indicating that the heterogeneity in study population did not impact our results.” (Page 27, lines 438-444)

**Title:** “**Factors associated with psychological distress during the coronavirus disease 2019 (COVID-19) pandemic on the predominantly general population: a systematic review and meta-analysis” (**Page 1, lines 1-3**)**

**S4 Table. Sensitivity analyses of factors and risks of anxiety and depression among studies excluding patients and healthcare professionals.**

|  | **Anxiety** | | | | **Depression** | | | |
| --- | --- | --- | --- | --- | --- | --- | --- | --- |
|  | **OR (95% CI)^a^** | ***N* of studies** | ***I*^2^ (%)** | **P for heterogeneity** | **RR (95% CI)^a^** | ***N* of studies** | ***I*^2^ (%)** | **P for heterogeneity** |
| **Gender (women vs. men)** |  |  |  |  |  |  |  |  |
| Overall results | 1.48 (1.29-1.71) | 29 | 90.8% | <0.001 | 1.16 (1.07-1.26) | 25 | 75.0% | <0.001 |
| Studies excluding patients and  healthcare professionals | 1.44 (1.25-1.67) | 26 | 90.6% | <0.001 | 1.12 (1.04-1.22) | 21 | 71.3% | <0.001 |
| **Age (younger vs. older)** |  |  |  |  |  |  |  |  |
| Overall results | 1.20 (1.13-1.26) | 21 | 91.7% | <0.001 | 1.13 (1.08-1.18) | 18 | 95.1% | <0.001 |
| Studies excluding patients and  healthcare professionals | 1.30 (1.21-1.41) | 19 | 92.3% | <0.001 | 1.20 (1.13-1.28) | 16 | 95.5% | <0.001 |
| **Education (lower vs. higher)** |  |  |  |  |  |  |  |  |
| Overall results | 1.21 (1.05-1.40) | 18 | 86.1% | <0.001 | 1.15 (1.03-1.29) | 20 | 82.0% | 0.016 |
| Studies excluding patients and  healthcare professionals | 1.18 (1.02-1.37) | 16 | 87.4% | <0.001 | 1.14 (1.01-1.28) | 17 | 84.3% | <0.001 |
| **Income (lower vs. higher)** |  |  |  |  |  |  |  |  |
| Overall results | 1.45 (1.24-1.69) | 13 | 82.3% | <0.001 | 1.56 (1.26-1.92) | 10 | 85.4% | <0.001 |
| Studies excluding patients and  healthcare professionals | 1.45 (1.24-1.70) | 13 | 82.3% | <0.001 | 1.56 (1.26-1.92) | 10 | 85.4% | <0.001 |
| **Current employment (yes vs. no)** |  |  |  |  |  |  |  |  |
| Overall results | 0.89 (0.78-1.02) | 8 | 26.6% | 0.22 | 0.76 (0.61-0.95) | 7 | 63.8% | 0.011 |
| Studies excluding patients and  healthcare professionals | 0.89 (0.78-1.02) | 8 | 26.6% | 0.22 | 0.76 (0.61-0.95) | 7 | 63.8% | 0.011 |
| **Residential area (rural vs. urban)** |  |  |  |  |  |  |  |  |
| Overall results | 1.13 (1.00-1.29) | 7 | 82.9% | <0.001 | 0.98 (0.85-1.12) | 7 | 81.6% | <0.001 |
| Studies excluding patients and  healthcare professionals | 1.13 (1.00-1.29) | 7 | 82.9% | <0.001 | 0.98 (0.85-1.12) | 7 | 81.6% | <0.001 |
| **Suspected/confirmed COVID-19 cases (yes vs. no)** | |  |  |  |  |  |  |  |
| Overall results | 1.70 (1.41-2.06) | 15 | 79.5% | <0.001 | 1.84 (1.39-2.43) | 8 | 73.5% | <0.001 |
| Studies excluding patients and  healthcare professionals | 1.71 (1.41-2.07) | 14 | 81.0% | <0.001 | 1.79 (1.34-2.38) | 7 | 75.9% | <0.001 |
| **Living in the hard-hit area (yes vs. no)** | |  |  |  |  |  |  |  |
| Overall results | 1.57 (1.36-1.81) | 10 | 73.9% | <0.001 | 1.33 (1.16-1.53) | 10 | 69.1% | 0.001 |
| Studies excluding patients and  healthcare professionals | 1.64 (1.43-1.87) | 9 | 67.0% | 0.002 | 1.32 (1.12-1.54) | 9 | 72.5% | <0.001 |
| **Pre-existing physical conditions (yes vs. no)** | |  |  |  |  |  |  |  |
| Overall results | 1.48 (1.21-1.81) | 11 | 65.2% | 0.001 | 1.42 (1.12-1.80) | 11 | 89.0% | <0.001 |
| Studies excluding patients and  healthcare professionals | 1.47 (1.17-1.85) | 10 | 68.2% | 0.001 | 1.38 (1.06-1.81) | 10 | 89.9% | <0.001 |
| **Pre-existing mental conditions (yes vs. no)** | |  |  |  |  |  |  |  |
| Overall results | 1.82 (1.34-2.48) | 6 | 70.8% | 0.004 | 1.75 (0.98-3.14) | 6 | 93.5% | <0.001 |
| Studies excluding patients and  healthcare professionals | 1.84 (1.30-2.61) | 4 | 78.7% | 0.003 | 1.74 (0.85-3.54) | 4 | 96.0% | <0.001 |
| **Media exposure (longer vs. shorter)** | |  |  |  |  |  |  |  |
| Overall results | 1.57 (1.16-2.13) | 6 | 94.5% | <0.001 | 1.34 (1.12-1.60) | 8 | 86.2% | <0.001 |
| Studies excluding patients and  healthcare professionals | 1.60 (1.14-2.27) | 5 | 95.4% | <0.001 | 1.34 (1.10-1.64) | 7 | 87.3% | <0.001 |
| **Social/family support (yes vs. no)** |  |  |  |  |  |  |  |  |
| Overall results | 0.68 (0.58-0.79) | 4 | 0% | 0.58 | 0.47 (0.40-0.56) | 5 | 0% | 0.50 |
| Studies excluding patients and  healthcare professionals | 0.68 (0.58-0.79) | 4 | 0% | 0.58 | 0.47 (0.40-0.56) | 5 | 0% | 0.50 |
| **Physical activity (longer vs. shorter)** | |  |  |  |  |  |  |  |
| Overall results | 0.71 (0.58-0.88) | 5 | 52.3% | 0.08 | 0.69 (0.50-0.94) | 6 | 84.8% | <0.001 |
| Studies excluding patients and  healthcare professionals | 0.71 (0.58-0.88) | 5 | 52.3% | 0.08 | 0.69 (0.50-0.94) | 6 | 84.8% | <0.001 |

**Abbreviations:** OR, odds ratio; CI, confidence interval.

^a^The odds ratios were calculated using the random-effects model.

*6. In terms of improving the quality of the Discussion, see point #4 above.*

**Response:** Thank you very much for your comments in improving the quality of the Discussion. As we mentioned in your response to your #4 comment, we have now further included all the updated and relevant meta-analyses in our manuscript, and further highlighted our novelty, as well as the rationale of our study in both Introduction and Discussion.

**Introduction:** “Although a few meta-analyses have been conducted to investigate the prevalence of psychological distress related to the COVID-19 pandemic, studies on determinants of psychological distress have largely focused on healthcare workers [6, 23-26]. Systematic reviews on factors associated with psychological distress in the general population during the COVID-19 pandemic have not been reported.” (Page 4, lines 62-67)

**Discussion:** “Previous meta-analyses among general population only reported the prevalence of psychological distress [6, 32-37]. In comparison, the primary objective of the current meta-analysis was to determine the factors associated with psychological distress using quantitative assessment during the COVID-19 pandemic in the predominantly general population. We are not aware of other meta-analyses assessing factors of psychological distress in the general population.” (Pages 26-27, lines 422-427)

**We have now highlighted the following under limitations of the study:** “Second, we included a predominantly general population for the current meta-analysis, with a few studies among high-risk and vulnerable patients, and a small subset of healthcare workers among studies using random sampling techniques, which may bring potential heterogeneity to our results. Nevertheless, we conducted sensitivity analyses and repeated all analyses among studies only containing general populations, and found the same significant results across all factors, indicating that the heterogeneity in study population did not impact our results.” (Page 27, lines 438-444)

***Reviewer #2:***

*The review explores the prevalence of mental health difficulties during the Covid-19 pandemic and the factors that are associated with such outcomes. The review has relevance and can make a contribution to the literature. My main issues are that the discussion should acknowledge weaknesses in the data and analysis. Principally that country of residence or nationality has not been included as a factor and that the mental health outcomes cannot be reliably attributed to Covid-19 from the data. I'd like to see the authors take up these issues and deal with them robustly in the discussion. More detailed comments are seen below.*

*1. There is no analysis of international region, surely there is variability here in the extent to which mental health difficulties arise given the wide variation in political and economic conditions in the many countries affected by Covid-19. Why did the authors not think that this was an important factor to consider in their analyses?*

**Response:** Thank you for your comments, we agree with the reviewer that different study locations may bring potential heterogeneity to our results, and we have now further added the stratified analyses and meta-regression according to study locations to explore potential heterogeneity. As a result, we found that most of associations had two or three available subgroups for the analysis, and the point estimate of odds ratio observed in the overall population was largely consistent across all subgroups, albeit the 95% CI became wider for subgroups with fewer studies, and did not achieve statistical significance for some groups with very few studies. However, except for the association of anxiety with gender (*P* for meta-regression=0.037), no statistically significant differences were found across subgroups of study locations (all *P*-values from meta-regression ≥0.09), indicating that geographic differences were less likely to influence the observed associations in the current meta-analysis. Nevertheless, we are aware that the small sample sizes and few numbers of subgroups for stratified analyses are limitations for the current study, and we have acknowledged them in the discussion. We have now added aforementioned changes in the manuscript, and also shown them below:

**Methods:** “The between-study heterogeneity was evaluated with Cochrane Q statistic (*P*<0.10 indicates statistical significance) and *I*^2^ statistic (>50% indicates possible heterogeneity) [41, 42]. We conducted meta-regression (*P* <0.05 indicates statistical significance) and stratified analyses by study locations … to evaluate the potential influence of geographic differences … on the results.” (Page 7, lines 128-133)

**Results:** “**Assessment of heterogeneity.** We observed a substantial heterogeneity for most of the associations between studies (*I^2^* range: 52.3%-95.1%), except for the association between current employment and anxiety (*I^2^*: 26.6%; *P*=0.22) and the associations of family/social support with both anxiety (*I^2^*: 0%; *P*=0.58) and depression (*I^2^*: 0%; *P*=0.58). We further conducted stratified analyses to explore the heterogeneity. We found that the point estimate of OR observed in the overall population was largely consistent across all subgroups by locations … with three or more studies, albeit the 95% CI became wider for subgroups with fewer studies, and did not achieve statistical significance for some groups with very few studies (S2 Table & S3 Table). Moreover, except for the association of anxiety with gender ((higher odds in studies in Asian than in Europe, *P* for meta-regression=0.037), no statistically significant differences were found across subgroups of study locations for other factors (all *P*-values from meta-regression ≥0.09), indicating that geographic differences were less likely to influence the observed associations in the current meta-analysis (S2 Table).” (Page 21, lines 288-300)

**Discussions:** “In addition, we conducted meta-regression and stratified analyses by study locations … and observed largely non-significant *P*-values for meta-regression analyses and consistent point estimates of OR across subgroups, suggesting no substantial statistical differences across subgroups. Nevertheless, the sample sizes and numbers of subgroups were small for the stratified analyses, thus the 95% CI became wider and did not achieve statistical significance for subgroups with very few studies; future studies with larger sample sizes are warranted to validate our findings.” (Page 28, lines 457-464)

**S2 Table. Stratified analyses of factors and risks of anxiety and depression by study locations.**

|  | **Anxiety** | | | |  | **Depression** | | | |  |
| --- | --- | --- | --- | --- | --- | --- | --- | --- | --- | --- |
|  | **OR (95% CI)^a^** | ***N* of studies** | ***I*^2^ (%)** | **P for heterogeneity** | **P for meta-regression** | **RR (95% CI)^a^** | ***N* of studies** | ***I*^2^ (%)** | **P for heterogeneity** | **P for meta-regression** |
| **Gender (women vs. men)** | |  |  |  |  |  |  |  |  |  |
| Overall results | 1.48 (1.29-1.71) | 29 | 90.8% | <0.001 |  | 1.16 (1.07-1.26) | 25 | 75.0% | <0.001 |  |
| Stratified by locations |  |  |  |  | 0.037 |  |  |  |  | 0.98 |
| Asia | 1.26 (1.01-1.57) | 11 | 90.6% | <0.001 |  | 1.11 (0.96-1.29) | 15 | 84.6% | <0.001 |  |
| Middle East | - | 2 | - | - |  | - | 2 | - | - |  |
| Europe | 1.12 (0.93-1.34) | 4 | 15.4% | 0.14 |  | - | 2 | - | - |  |
| U.S. | - | 1 | - | - |  | - | 0 | - | - |  |
| **Age (younger vs. older)** | |  |  |  |  |  |  |  |  |  |
| Overall results | 1.20 (1.13-1.26) | 21 | 91.7% | <0.001 |  | 1.13 (1.08-1.18) | 18 | 95.1% | <0.001 |  |
| Stratified by locations |  |  |  |  | 0.87 |  |  |  |  | 0.40 |
| Asia | 1.36 (1.19-1.56) | 13 | 91.3% | <0.001 |  | 1.25 (1.12-1.40) | 13 | 95.9% | <0.001 |  |
| Middle East | 1.23 (0.86-1.76) | 3 | 76.7% | - |  | - | 2 | - | - |  |
| Europe | 1.10 (1.03-1.18) | 4 | 93.3% | <0.001 |  | 1.02 (0.99-1.06) | 3 | 89.8% | <0.001 |  |
| U.S. | - | 0 | - | - |  | - | 0 | - | - |  |
| **Education (lower vs. higher)** | |  |  |  |  |  |  |  |  |  |
| Overall results | 1.21 (1.05-1.40) | 18 | 86.1% | <0.001 |  | 1.15 (1.03-1.29) | 20 | 82.0% | 0.016 |  |
| Stratified by locations |  |  |  |  | 0.81 |  |  |  |  | 0.27 |
| Asia | 1.26 (1.01-1.57) | 11 | 90.6% | <0.001 |  | 1.11 (0.96-1.29) | 15 | 84.6% | <0.001 |  |
| Middle East | - | 2 | - | - |  | - | 2 | - | - |  |
| Europe | 1.12 (0.93-1.34) | 4 | 45.4% | 0.14 |  | - | 2 | - | - |  |
| U.S. | - | 1 | - | - |  | - | 1 | - | - |  |
| **Income (lower vs. higher)** | |  |  |  |  |  |  |  |  |  |
| Overall results | 1.45 (1.24-1.69) | 13 | 82.3% | <0.001 |  | 1.56 (1.26-1.92) | 10 | 85.4% | <0.001 |  |
| Stratified by locations |  |  |  |  | 0.69 |  |  |  |  | 0.62 |
| Asia | 1.46 (1.20-1.78) | 9 | 87.3% | <0.001 |  | 1.47 (1.12-1.91) | 7 | 89.0% | <0.001 |  |
| Middle East | - | 1 | - | - |  | - | 1 | - | - |  |
| Europe | - | 1 | - | - |  | - | 1 | - | - |  |
| U.S. | - | 2 | - | - |  | - | 1 | - | - |  |
| **Current employment (yes vs. no)** | |  |  |  |  |  |  |  |  |  |
| Overall results | 0.89 (0.78-1.02) | 8 | 26.6% | 0.22 |  | 0.76 (0.61-0.95) | 7 | 63.8% | 0.011 |  |
| Stratified by locations |  |  |  |  | 0.31 |  |  |  |  | 0.42 |
| Asia | 0.87 (0.76-1.00) | 4 | 0% | 0.48 |  | 0.95 (0.61-1.49) | 3 | 80% | 0.007 |  |
| Middle East | - | 2 | - | - |  | - | 2 | - | - |  |
| Europe | - | 1 | - | - |  | - | 1 | - | - |  |
| U.S. | - | 1 | - | - |  | - | 1 | - | - |  |
| **Residential area (rural vs. urban)** | |  |  |  |  |  |  |  |  |  |
| Overall results | 1.13 (1.00-1.29) | 7 | 82.9% | <0.001 |  | 0.98 (0.85-1.12) | 7 | 81.6% | <0.001 |  |
| Stratified by locations |  |  |  |  | 0.11 |  |  |  |  | 0.15 |
| Asia | 1.11 (0.98-1.25) | 6 | 83.2% | <0.001 |  | 0.95 (0.83-1.08) | 6 | 81.9% | <0.001 |  |
| Middle East | - | 0 | - | - |  | - | 0 | - | - |  |
| Europe | - | 1 | - | - |  | - | 1 | - | - |  |
| U.S. | - | 0 | - | - |  | - | 0 | - | - |  |
| **Suspected/confirmed COVID-19 cases (yes vs. no)** | | |  |  |  |  |  |  |  |  |
| Overall results | 1.70 (1.41-2.06) | 15 | 79.5% | <0.001 |  | 1.84 (1.39-2.43) | 8 | 73.5% | <0.001 |  |
| Stratified by locations |  |  |  |  | 0.66 |  |  |  |  | 0.58 |
| Asia | 1.91 (1.50-2.42) | 10 | 60.7% | 0.006 |  | 1.98 (1.47-2.67) | 6 | 57.9% | 0.036 |  |
| Middle East | - | 2 | - | - |  | - | 1 | - | - |  |
| Europe | - | 2 | - | - |  | - | 1 | - | - |  |
| U.S. | - | 1 | - | - |  | - | 0 | - | - |  |
| **Living in the hard-hit area (yes vs. no)** | |  |  |  |  |  |  |  |  |  |
| Overall results | 1.57 (1.36-1.81) | 10 | 73.9% | <0.001 |  | 1.33 (1.16-1.53) | 10 | 69.1% | 0.001 |  |
| Stratified by locations |  |  |  |  | 0.47 |  |  |  |  | 0.68 |
| Asia | 1.61 (1.38-1.88) | 9 | 73.0% | <0.001 |  | 1.31 (1.11-1.54) | 9 | 72.4% | <0.001 |  |
| Middle East | - | 0 | - | - |  | - | 0 | - | - |  |
| Europe | - | 0 | - | - |  | - | 0 | - | - |  |
| U.S. | - | 1 | - | - |  | - | 1 | - | - |  |
| **Pre-existing physical conditions (yes vs. no)** | |  |  |  |  |  |  |  |  |  |
| Overall results | 1.48 (1.21-1.81) | 11 | 65.2% | 0.001 |  | 1.42 (1.12-1.80) | 11 | 89.0% | <0.001 |  |
| Stratified by locations |  |  |  |  | 0.09 |  |  |  |  | 0.11 |
| Asia | - | 2 | - | - |  | 1.66 (1.12-2.45) | 5 | 93.7% | <0.001 |  |
| Middle East | 0.98 (0.63-1.53) | 3 | 29.9% | 0.24 |  | - | 2 | - | - |  |
| Europe | 1.60 (1.19-2.14) | 5 | 68.3% | 0.013 |  | 1.44 (1.04-1.98) | 4 | 75.4% | 0.007 |  |
| U.S. | - | 1 | - | - |  | - | 0 | - | - |  |
| **Pre-existing mental conditions (yes vs. no)** | |  |  |  |  |  |  |  |  |  |
| Overall results | 1.82 (1.34-2.48) | 6 | 70.8% | 0.004 |  | 1.75 (0.98-3.14) | 6 | 93.5% | <0.001 |  |
| Stratified by locations |  |  |  |  | 0.13 |  |  |  |  | 0.38 |
| Asia | - | 2 | - | - |  | - | 2 | - | - |  |
| Middle East | 1.57 (0.90-2.73) | 3 | 68.0% | 0.044 |  | 1.34 (0.75-2.41) | 3 | 83.1% | 0.003 |  |
| Europe | - | 0 | - | - |  | - | 0 | - | - |  |
| U.S. | - | 1 | - | - |  | - | 1 | - | - |  |
| **Media exposure (longer vs. shorter)** | |  |  |  |  |  |  |  |  |  |
| Overall results | 1.57 (1.16-2.13) | 6 | 94.5% | <0.001 |  | 1.34 (1.12-1.60) | 8 | 86.2% | <0.001 |  |
| Stratified by locations |  |  |  |  | 0.17 |  |  |  |  | 0.47 |
| Asia | 1.80 (1.36-2.38) | 4 | 82.9% | 0.001 |  | 1.43 (1.13-1.80) | 6 | 79.4% | <0.001 |  |
| Middle East | - | 0 | - | - |  | - | 0 | - | - |  |
| Europe | - | 2 | - | - |  | - | 2 | - | - |  |
| U.S. | - | 0 | - | - |  | - | 0 | - | - |  |
| **Social/family support (yes vs. no)** | |  |  |  |  |  |  |  |  |  |
| Overall results | 0.68 (0.58-0.79) | 4 | 0% | 0.58 |  | 0.47 (0.40-0.56) | 5 | 0% | 0.50 |  |
| Stratified by locations |  |  |  |  | 0.89 |  |  |  |  | 0.42 |
| Asia | 0.69 (0.57-0.83) | 3 | 0% | 0.54 |  | 0.43 (0.34-0.55) | 4 | 0% | 0.54 |  |
| Middle East | - | 0 | - | - |  | - | 0 | - | - |  |
| Europe | - | 0 | - | - |  | - | 0 | - | - |  |
| U.S. | - | 1 | - | - |  | - | 1 | - | - |  |
| **Physical activity (longer vs. shorter)** | |  |  |  |  |  |  |  |  |  |
| Overall results | 0.71 (0.58-0.88) | 5 | 52.3% | 0.08 |  | 0.69 (0.50-0.94) | 6 | 84.8% | <0.001 |  |
| Stratified by locations |  |  |  |  | - |  |  |  |  | - |
| Asia | 0.71 (0.58-0.88) | 5 | 52.3% | 0.08 |  | 0.69 (0.50-0.94) | 6 | 84.8% | <0.001 |  |
| Middle East | - | 0 | - | - |  | - | 0 | - | - |  |
| Europe | - | 0 | - | - |  | - | 0 | - | - |  |
| U.S. | - | 0 | - | - |  | - | 0 | - | - |  |

**Abbreviations:** OR, odds ratio; CI, confidence interval.

^a^The odds ratios were calculated using the random-effects model among studies with 4 or more data points.

*2. The measures used in the studies were mainly self-report questionnaires and this raises the possibility of demand characteristics and over-reporting of symptoms: what do the authors have to say about these potential confounds? The results suggest that 1 in 3 respondents have anxiety or depression, this is consistent with the Luo et al. (2020) meta-analysis as you say, but their review sampled studies which used the same types of self-report measures. This does not invalidate the reviews but it does present a very real weakness to claims about prevalence.*

**Response:** Thank you for your comments. We agree with the reviewer that the self-reported questionnaires may bring potential bias and the likelihood of over-reporting symptoms, which is a limitation that would impact the prevalence of psychological distress reported in the current study. However, in terms of the associations between factors and psychological distress, this could lead to non-differential misclassification and result in an underestimation of the true effect size of factors with psychological distress. For example, when assessing the association between age and anxiety, people with older age and younger age are likely to have the same prevalence of over-reporting of symptoms. Therefore, the significant associations observed in the current study are not likely to be impact by self-reported questionnaires. We have now added the changes to the manuscript:

**Discussion:** “Third, psychological distress was measured using self-reported questionnaires, which may have brought bias to an overestimation or underestimation of the true prevalence of psychological distress. However, for associations between factors and psychological distress, self-reported bias would lead to non-differential misclassifications and result in an underestimation of the true effect size of factors and psychological distress. Therefore, the significant associations between factors and psychological distress observed in the current study is unlikely to be impacted by self-reported questionnaires.” (Pages 27-28, lines 444-451)

*3. On page 22, last paragraph, you make the point about how age effects could be moderated by previous experience of infectious outbreaks. This is another reason why incorporating respondents’ country of residence is important, as some countries in Asia for example will have already experienced the SARS virus and might have better mental health outcomes as a result of this.*

**Response:** Thank you for your comments, as we mentioned in the response to your first comment, we have now added the stratified analysis according to the study locations. For age specifically, we had 21 studies assessing age-anxiety association, and 18 studies assessing age-depression association, and the odds ratio were 1.20 (1.13-1.26) and 1.13 (1.08-1.18), respectively. In the stratified analyses, the point estimate for odds ratio among Asia, Middle East, and Europe were essentially the same, and there was no study assessing this association in the US. The meta-regression was non-significant for age with anxiety and depression across subgroups (*P* for meta-regression =0.87 and 0.40, respectively), indicating that the geographic differences were less likely to influence the observed association between age and psychological distress in the current meta-analysis. Please kindly refer to our response to your first comment for changes in the manuscript.

*4. The discussion point raised on page 23 about how rural areas have poorer healthcare infrastructure, economy, sanitation, and education is contentious. In many countries the reverse might be true, whereby urban areas offer poorer environments/infrastructure.*

**Response:** Thank you for your comment. We agree with the reviewer that the reverse might also be true that urban areas had poorer environment and infrastructure, which was supported by a recent policy brief published by the United Nation. We have now added this discussion in the manuscript:

**Discussion:** “However, we found higher odds of anxiety among rural versus urban dwellers in our analysis, albeit the association with depression was not significant. As rural areas during the COVID-19 pandemic may be a reflection of the poorer healthcare infrastructure, economy, sanitation, and educational resources [19], the observed rural-urban gradient has important public health implication of ensuring equitable healthcare resources in rural and resource-restraint areas during the COVID-19 pandemic, and further studies are warranted to investigate rural-depression association. Nevertheless, according to the United Nations, in many countries, the reverse might be true, whereby urban areas may have poorer environment and infrastructure for the prevention of COVID-19 [121]. In the current meta-analysis, we had relatively small sample size for the association between residential area and psychological distress (nine studies for anxiety and depression), and further studies with larger sample sizes and higher statistical power are warranted to examine this association.” (Page 25, lines 386-398)

*5. The paper reports prevalence of mental health outcomes, but there is no way of linking such outcomes to the effects of Covid-19 itself. How do the authors address this issue and how does it affect the confidence of the conclusions they draw?*

**Response:** Thank you for your comments. We agree that it is important to clarify the prevalence of psychological distress with the effects of COVID-19 itself. In the current meta-analysis, we calculated the attributable risk of psychological distress due to COVID-19 to measure the risk under exposure. As a result, we had available data from two studies reported the prevalence of depression among patients with and without COVID-19 from China and Vietnam at the start of the outbreak in February 2020. The prevalence of COVID-19 infection at that time was much higher in China compared to Vietnam (0.56 per 10,000 people vs. 0.0017 per 10,000 people). Therefore, the prevalence of depression among patients with and without COVID-19 in China (51.6%	and 41.9%) was higher than that in Vietnam (13.6% and 7.04%). However, the calculated attributable risk of depression due to COVID-19 was similar in these two studies (9.70% vs. 9.52%). Of note, the prevalence of depression among COVID-19 patients observed in the Chinese study was similar to the result reported in a recent meta-analysis among COVID-19 patients (45% [95% CI: 37%-54%]) (Deng J et al. The prevalence of depression, anxiety, and sleep disturbances in COVID-19 patients: a meta-analysis. Ann N Y Acad Sci

. 2020 Oct 2;10.1111/nyas.14506.). As the impact of COVID-19 has become substantially wider globally, the attributable risk is likely to have increased as the pandemic evolves. We have now added the discussion in the manuscript:

**Methods:** “If included studies reported the prevalence of psychological distress among patients with and without COVID-19, we further calculated the attributable risk of psychological distress due to COVID-19 by the formula $\frac{{R1}/{R0}-1}{R1/R0}$, where ${R1}/{R0}$is the causal risk ratio that measures the risk under exposure (COVID-19) [19].” (Page 8, lines 150-153)

**Results:** “**Attributable risk.** Two studies (one from China and one from Vietnam) reported the prevalence of depression among patients with and without COVID-19 [70, 81]; the prevalence of depression among patients with COVID-19 was 51.6% in China and 13.6% in Vietnam. The prevalence of depression among patients without COVID-19 was 41.9% in China and 7.04% in Vietnam. The attributable risk of depression due to the COVID-19 pandemic was 9.70% and 9.52% from the study conducted in China and Vietnam, respectively (S5 Table).” (Page 22, lines 318-323)

**Discussion:** “We also observed similar attributable risk of psychological distress due to COVID-19 (around 10%) at the start of the outbreak in February 2020 from two studies (from China [81] and Vietnam [70]) with available data, albeit the prevalence of depression among patients with and without COVID-19 was higher in China (51.6% and 41.9%) compared to that in Vietnam (13.6% and 7.04%) possibility due to the higher infection rate in China (0.56 per 10,000 people [103] vs. 0.0017 per 10,000 people in Vietnam [104]). The prevalence of depression among COVID-19 patients observed in the Chinese study was similar to that reported in a recent meta-analysis among COVID-19 patients (45% [95% CI: 37%-54%]) [36]. As the impact of COVID-19 has become substantially wider globally, the attributable risk is likely to have increased as the pandemic evolves.” (Page 23, lines 341-350)

**S5 Table.** **The attributable risk of depression due to the COVID-19 pandemic.**

| **Author, study location** | **Study period** | **Prevalence of COVID-19 infection** | **Prevalence of depression among patients with COVID-19** | **Prevalence of depression among participants without COVID-19** | **Attributable risk of depression due to the COVID-19 pandemic^a^** |
| --- | --- | --- | --- | --- | --- |
| Ma, China^62^ | February 24 and March 8, 2020 | 0.56 per 10,000 people^69,70^ | 51.6% | 41.9% | 9.70% |
| Nguyen, Vietnam^13^ | February 14 and March 2, 2020 | 0.0017 per 10,000 people^69,71^ | 13.6% | 7.04% | 9.52% |

^a^The attributable risk of depression due to the COVID-19 pandemic was calculated by the formula $\frac{{R1}/{R0}-1}{R1/R0}$, where ${R1}/{R0}$ is the causal risk ratio that measures the risk under exposure (COVID-19).^72^
